# Aptamer‐Functionalized Nanocarriers for Bone and Joint Diseases: Engineering Design Rules and Translational Roadmap

**DOI:** 10.1002/smsc.70294

**Published:** 2026-05-11

**Authors:** Hamdi Al‐Azzani, Djandan Tadum Arthur Vithran, Syeda Sundas Batool, Malek Mohammed Ali Alshabi, Wenhu Zhou, Xiaoqiang Mao

**Affiliations:** ^1^ Xiangya School of Pharmaceutical Sciences Central South University Changsha China; ^2^ Department of Orthopedics Xiangya Hospital Central South University Changsha China; ^3^ National Clinical Research Center for Geriatric Disorders Xiangya Hospital Central South University Changsha China; ^4^ Department of Microbiology School of Basic Medical Sciences Central South University Changsha China; ^5^ Department Orthopedic Xiangya Third Hospital Changsha China; ^6^ Hunan Key Laboratory of the Research and Development of Novel Pharmaceutical Preparations School of Pharmaceutical Science Changsha Medical University Changsha China; ^7^ The Quzhou Affiliated Hospital of Wenzhou Medical University Quzhou People's Hospital Quzhou China

**Keywords:** aptamers, bone regeneration, DNA nanostructures, intraarticular therapy, musculoskeletal diseases, nanocarriers, osteoarthritis, targeted drug delivery

## Abstract

The escalating burden of musculoskeletal disorders, such as osteoarthritis, osteoporosis, inflammatory arthritis, bone tumors, and skeletal infections, necessitates precisely targeted therapeutics beyond conventional interventions. Nucleic acid aptamers are a next‐generation ligand class distinguished by their high affinity, target specificity, low immunogenicity, and programable chemical properties. Their incorporation into nanoparticles, DNA nanostructures, hydrogels, microneedles, exosomes, and implant coatings is reshaping the therapeutic landscape of bone and joint diseases. This review synthesizes preclinical evidence from in vivo and ex vivo models, highlighting aptamer‐functionalized carriers for targeting bone resorption, cartilage damage, synovial inflammation, bacterial infections, and skeletal malignancies. A classification framework based on aptamer target types, including cellular, extracellular matrix, signaling pathway, and pathogen‐specific ligands, is mapped to appropriate nanocarriers and delivery routes. Key engineering parameters, including dissociation constant, ligand density, multivalency, linker design, particle size, and surface charge, are critical determinants of biodistribution, tissue penetration, and target specificity. Bone‐to‐reticuloendothelial system ratio and joint tissue retention metrics were proposed to guide rational design. Safety profiles, immunogenicity, and manufacturing feasibility were integrated into a translational roadmap. Standardized reporting protocols and priority indications, including intraarticular delivery in arthritis, bone defect regeneration, and targeted delivery in bone malignancies, have been identified to facilitate clinical translation.

## Graphical abstract

Aptamer‐functionalized nanocarriers represent a versatile platform for targeted therapy in bone and joint diseases. This review presents five complementary design strategies: free aptamers for soluble targets, bispecific multivalent constructs for enhanced avidity, nanoparticle‐conjugated aptamers for selective tissue accumulation, aptamer‐based molecular switches for stimulus‐responsive activity, and scaffold‐integrated aptamers for spatially confined local delivery within musculoskeletal defects.

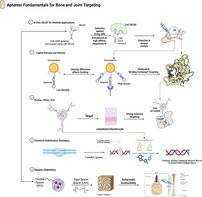

## 1 Introduction

Musculoskeletal disorders, including osteoarthritis, osteoporosis, inflammatory arthritides, bone tumors, and skeletal infections, represent a growing public health burden in the aging global population. Osteoarthritis affects up to 50% of adults aged ≥51 years and causes pain and disability [1]. Osteoporotic fractures are associated with elevated mortality, institutionalization, and long‐term health needs [2, 3, 4, 5]. Inflammatory diseases, such as rheumatoid arthritis (RA) and spondyloarthritis, cause irreversible joint damage and systemic complications, severely impacting quality of life [6, 7, 8]. Malignant bone tumors and skeletal metastases further compromise structural integrity and pose treatment challenges.

Collectively, musculoskeletal conditions affected approximately 1.69 billion individuals worldwide in 2021, representing a 95% increase since 1990, and remain among the leading contributors to global disability [9]. The annual economic burden of osteoarthritis in the United States is estimated at approximately 136.8 billion dollars in combined direct medical costs and lost productivity [10, 11]. In the European Union, the costs associated with osteoporosis were estimated at 37 billion euros in 2017, with more than 70% attributed to fracture‐related expenses, while in the United States, the direct costs of osteoporotic fractures exceed 19 billion dollars annually [10, 11].

Current standard‐of‐care interventions, including systemic anti‐inflammatory drugs, bisphosphonates, and intraarticular corticosteroid injections, provide symptomatic relief but are limited by dose‐dependent toxicities, inadequate disease modification, and an inability to discriminate between distinct cellular and molecular targets within the musculoskeletal microenvironment. These unmet clinical needs underscore the importance of precisely targeted therapeutic strategies.

Despite pharmacologic advancements, drug delivery to bone and joint tissues remains poorly controlled. Systemically administered biologics and small molecules interact with heterogeneous targets in the subchondral bone, cartilage, marrow, and inflamed synovium; however, they lack sustained, localized exposure, which often leads to off‐target effects. Local intraarticular injections or depots offer transient concentration spikes; however, synovial vascularity and lymphatic drainage rapidly reduce residence time. Moreover, most delivery platforms fail to discriminate between specific cell types or extracellular compartments, resulting in a mismatch between drug complexity and delivery precision [12, 13, 14, 15].

Ligand‐directed nanocarriers offer a rational strategy to enhance the targeting of musculoskeletal compartments. Antibodies, peptides, small molecules, and bisphosphonates have been employed to functionalize drug carriers for improved localization in bone, cartilage, synovium, and tumor microenvironments [16, 17, 18, 19, 20]. However, antibodies are large and immunogenic; peptides and small molecules often lack specificity or exhibit poor pharmacokinetics; bisphosphonates bind minerals with high affinity but provide little control over cellular targeting and may disrupt remodeling [21, 22, 23, 24, 25, 26]. In contrast, aptamers are short nucleic acid ligands selected via systematic evolution of ligands by exponential enrichment. They combine high affinity and specificity with low immunogenicity, modularity, and ease of conjugation. Chemical modifications, such as 2′‐fluoro, 2′‐O‐methyl, and locked nucleic acids, enhance stability and have been successfully integrated into liposomes, polymeric nanoparticles, DNA nanostructures, hydrogels, microneedles, exosomes, and implant coatings [27, 28]. Pegaptanib's clinical success validated aptamer therapeutics in humans, and musculoskeletal applications have since expanded rapidly. Cell‐ and matrix‐binding aptamers have been developed against osteoblasts, mesenchymal stromal cells (MSCs), chondrocytes, synovial fibroblasts, sclerostin, connective tissue growth factor (CTGF), and tumor‐associated targets in osteosarcoma and bone metastases [28, 29].

However, no prior review has systematically integrated aptamer target discovery, carrier design, delivery route, biodistribution, therapeutic outcome, safety, and chemistry, manufacturing, and control considerations across the musculoskeletal disease spectrum. The existing literature lacks a quantitative framework for linking aptamer affinity, valency, spacer architecture, and nanoparticle properties to functional delivery outcomes. This concept‐based narrative review addresses this gap by analyzing in vivo and ex vivo models in which aptamers function as targeting ligands or therapeutic payloads within bone, cartilage, meniscus, synovium, marrow, or infected joint environments. Carrier systems include nanoparticles, scaffolds, DNA nanostructures, microneedles, hydrogels, exosomes, and implant coatings. The evaluated outcomes encompass bone mineral density (BMD), synovial inflammation, tumor suppression, and infection control. By synthesizing these findings, we propose a translational framework for aptamer‐guided nanocarriers in musculoskeletal precision therapy.

## 2 Aptamer Fundamentals for Bone and Joint Targeting

Aptamer‐functionalized nanocarriers enable a level of specificity in musculoskeletal targeting that is beyond the reach of most conventional therapeutics. Unlike small molecules and biologics that distribute indiscriminately throughout the body, aptamers can be engineered to recognize specific cell types, such as osteoblasts, chondrocytes, synovial fibroblasts, marrow stromal cells, tumor cells, or bacterial biofilms, while maintaining functional stability in nuclease‐rich and mechanically stressed environments, including bone, cartilage, synovium, and marrow. Their success depends on three interrelated parameters: the method of aptamer identification, their stabilization within skeletal tissues, and the optimization of affinity, ligand density, valency, and spacer architecture on carrier surfaces [30, 31, 32, 33].

### 2.1 Systematic Evolution of Ligands by Exponential Enrichment (SELEX), Cell‐SELEX, and In Vivo SELEX in Skeletal Microenvironments

One of the foundational techniques in aptamer discovery is SELEX, which enables the selection of high‐affinity oligonucleotides from libraries containing up to 10^15^ variants against a specific target molecule [34, 35, 36, 37, 38]. Traditional SELEX using purified proteins has produced aptamers against soluble factors and membrane receptors [35, 39]. However, in musculoskeletal tissues, critical epitopes are embedded in structurally complex environments, such as mineralized bone matrices, cartilage networks, or inflamed pannus tissue, where binding is influenced by architecture and pathology [40, 41]. This has prompted the evolution of cell‐SELEX, tissue‐SELEX, and in vivo SELEX techniques that preserve native conformations and posttranslational features while enabling selection under physiological barriers [42, 43, 44, 45, 46, 47].

**FIGURE 1:**
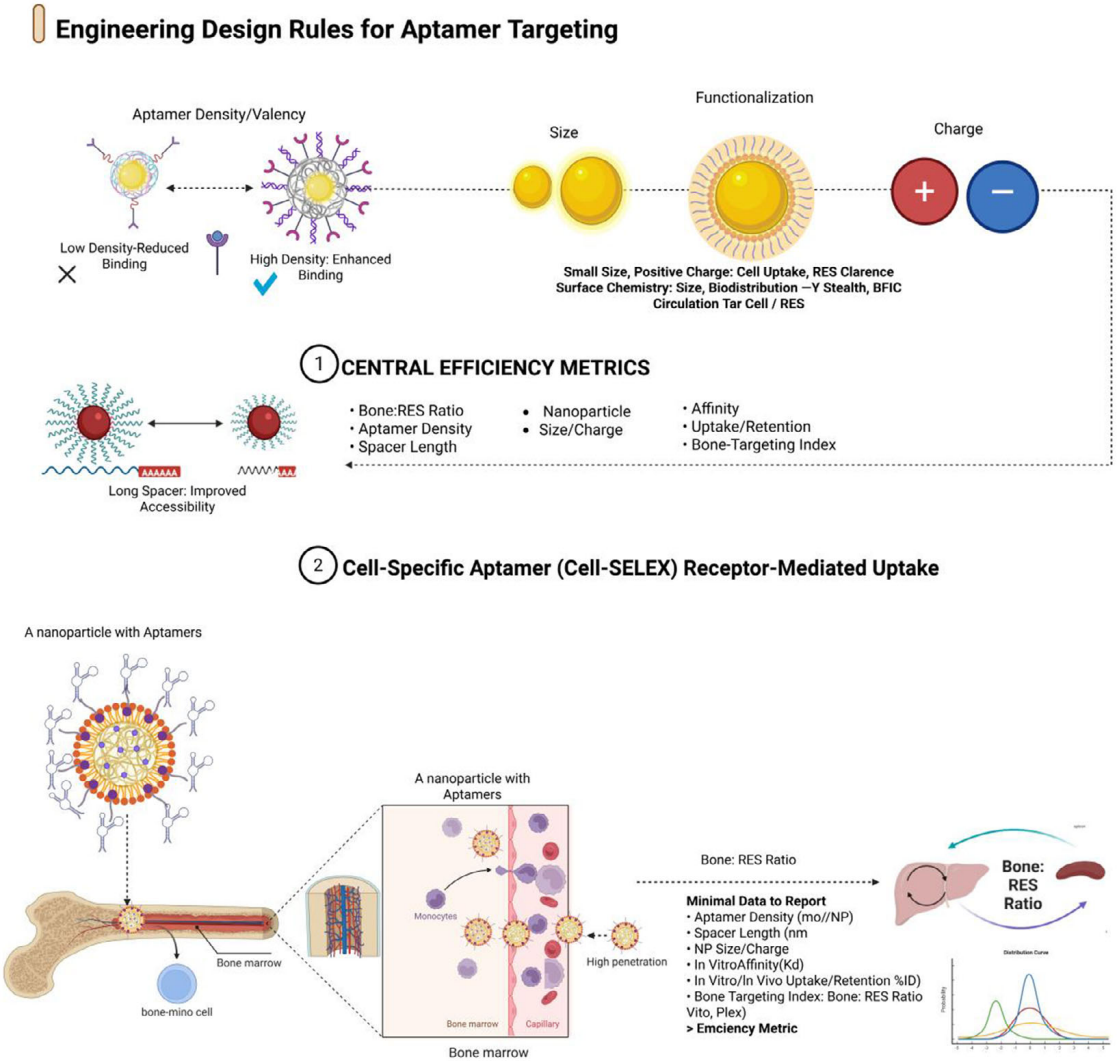
Engineering design rules for aptamer targeting and cell‐specific uptake. Design framework linking nanoscale parameters to aptamer performance in bone and joint tissues. The upper panels summarize the key engineering levers for surface presentation: aptamer density/valency (low versus high density and its impact on binding), carrier properties and chemistry (size, functionalization, and surface charge determining uptake, biodistribution, and reticuloendothelial system (RES) clearance), and spacer length (short versus long linkers controlling target accessibility and steric hindrance). The lower panel depicts cell‐SELEX‐derived, cell‐specific aptamers that bind to receptors on bone or marrow cells to drive receptor‐mediated uptake of aptamer‐decorated nanoparticles. The bone:RES ratio and minimal data elements to report, such as aptamer density, spacer length, nanoparticle size/charge, affinity, uptake/retention, and bone‐targeting index, are highlighted as the central efficiency metrics. Key examples include the CH6 osteoblast‐binding aptamer selected via cell‐SELEX [50], the MSC‐recruiting aptamer Apt19S used in meniscal scaffolds [52], and bone‐homing aptamers identified through in vivo SELEX [53].Created using BioRender.com.

Cell‐SELEX, which uses live cells for selection, has proven particularly effective for musculoskeletal targets [47, 48]. The osteoblast‐binding aptamer CH6, selected against osteoblast‐like cells, demonstrated nanomolar affinity and retained its targeting functionality when incorporated into tetrahedral DNA nanostructures embedded in GelMA hydrogels. In osteoporotic mandibular defects, CH6‐guided constructs promoted osteoblast recruitment and improved bone repair outcomes [49, 50]. Similarly, Apt19S was identified through stromal cell‐SELEX and incorporated into meniscal scaffolds, resulting in enhanced recruitment of MSCs and region‐specific fibrocartilage regeneration [51]. These examples show that aptamers derived from cell‐SELEX retain their targeting functionality even when conjugated to three‐dimensional biomaterials in dynamic joint environments (Figure 1).

In vivo SELEX advances this approach by applying selection pressure within living organisms, allowing aptamer pools to evolve under real pharmacokinetic constraints such as vascular transport, clearance, and tissue retention [39, 44]. Chen et al. (2018) used in vivo SELEX to isolate a bone‐homing DNA aptamer that showed enhanced skeletal accumulation when conjugated to nanoparticles compared to untargeted controls in a prostate cancer bone metastasis model [53]. Although this model was oncologically focused, the approach inherently addresses biological barriers, such as marrow filtration, systemic clearance, and tissue penetration. Similar selection strategies have produced aptamers that target skeletal muscle by enabling internalization and retention in vivo [54]. The evolution from protein‐based SELEX to cell and in vivo SELEX has expanded the aptamers toward ligands functionally conditioned for use in complex musculoskeletal environments, offering translational potential for conditions including chronic bone loss, osteomyelitis, and inflammatory arthritis [44, 47].

In addition to selection platform diversity, recent technological innovations have accelerated aptamer discovery and improved the quality of selected candidates. Microfluidic‐based SELEX miniaturizes incubation, partitioning, and amplification steps within integrated chip systems, enabling precise control over target concentration and washing stringency while dramatically reducing reagent consumption and selection rounds [55, 56]. Capillary electrophoresis SELEX separates target‐bound sequences from unbound pools based on electrophoretic mobility differences, allowing the identification of high‐affinity aptamers in as few as one to four selection rounds without the need for target immobilization^57^. Recently, Pro‐SELEX, a high‐dimensional microfluidic approach combining particle display, magnetic sorting, and high‐content bioinformatics, has demonstrated the ability to generate aptamers with programable binding affinities spanning a 20‐fold range within a single selection round [57]. In parallel, the integration of high‐throughput sequencing (HTS) with SELEX has enabled comprehensive monitoring of library enrichment dynamics across selection rounds, identifying high‐affinity candidates at earlier stages and reducing the need for extensive cloning and individual characterization [58]. These advances are particularly relevant for musculoskeletal targets, where complex tissue architecture and limited epitope accessibility have historically constrained selection efficiency. The application of microfluidic and sequencing‐coupled SELEX for cell‐ and tissue‐based selection against skeletal targets has the potential to substantially expand the available aptamer repertoire for bone and joint applications.

### 2.2 Chemical Stabilization Tailored to Bone, Cartilage, Synovium, and Marrow

Unmodified DNA and RNA aptamers are rapidly degraded by endonucleases and exonucleases in biological fluids, including plasma, serum, and synovial fluid, resulting in in vivo functional half‐lives that are often limited to minutes. Chemical stabilization is therefore essential for both systemically administered bone‐targeted constructs and locally delivered intraarticular depots [28, 47, 59, 60, 61]. Stabilization strategies have been applied to free aptamers as well as aptamers displayed on nanoparticles, DNA nanostructures, and hydrogels, enabling repeated or sustained exposure profiles in bone and joint disease models (Figure 1).

One widely adopted approach involves increasing structural rigidity through LNA, which constrains ribose conformation and stabilizes the secondary structure. When incorporated judiciously, LNA enhances nuclease resistance and may improve binding performance [59, 61].Backbone modifications, particularly phosphorothioate linkages, further improve resistance to enzymatic degradation and support stable conjugation to nanoparticle surfaces or scaffold backbones. However, these modifications can increase nonspecific protein interactions and introduce innate immune or complement‐related liabilities in certain oligonucleotide contexts [62, 63].

PEGylation remains the most established method for increasing hydrodynamic size, reducing renal clearance, and extending systemic circulation time, which is advantageous for delivery to skeletal tissues and bone‐associated lesions [28]. Nevertheless, pre‐existing or treatment‐induced anti‐PEG antibodies and PEG‐associated hypersensitivity reactions have been reported in humans, raising concerns for chronic musculoskeletal conditions that require long‐term or repeated administration, including osteoporosis, osteoarthritis, and RA [64, 65]. These limitations have stimulated interest in PEG alternatives, such as zwitterionic polymers, stealth‐like synthetic coatings, and hydrophilic polysaccharide‐based materials, which aim to preserve favorable pharmacokinetics while potentially reducing immunogenicity [66, 67, 68]. In addition to systemic clearance, musculoskeletal microenvironments impose additional stabilization challenges. In inflammatory arthritis, synovial fluid is enriched in proteases and nucleases that accelerate oligonucleotide degradation. Bone marrow niches further expose constructs to fluctuating pH, hypoxia, macrophage surveillance, and stromal cell uptake. Consequently, translationally oriented musculoskeletal aptamer systems frequently employ multilayered stabilization strategies that combine two‐prime sugar substitutions, carefully limited phosphorothioate incorporation, and appropriately sized stealth coatings. This integrated approach preserves aptamer folding and functional performance when deployed within hydrogels, extracellular vesicle‐like carriers, microneedles, or solid implant platforms [28, 61, 69].

### 2.3 Design Variables: Affinity, Ligand Density/Valency, Spacer, and Linker Chemistry

**FIGURE 2:**
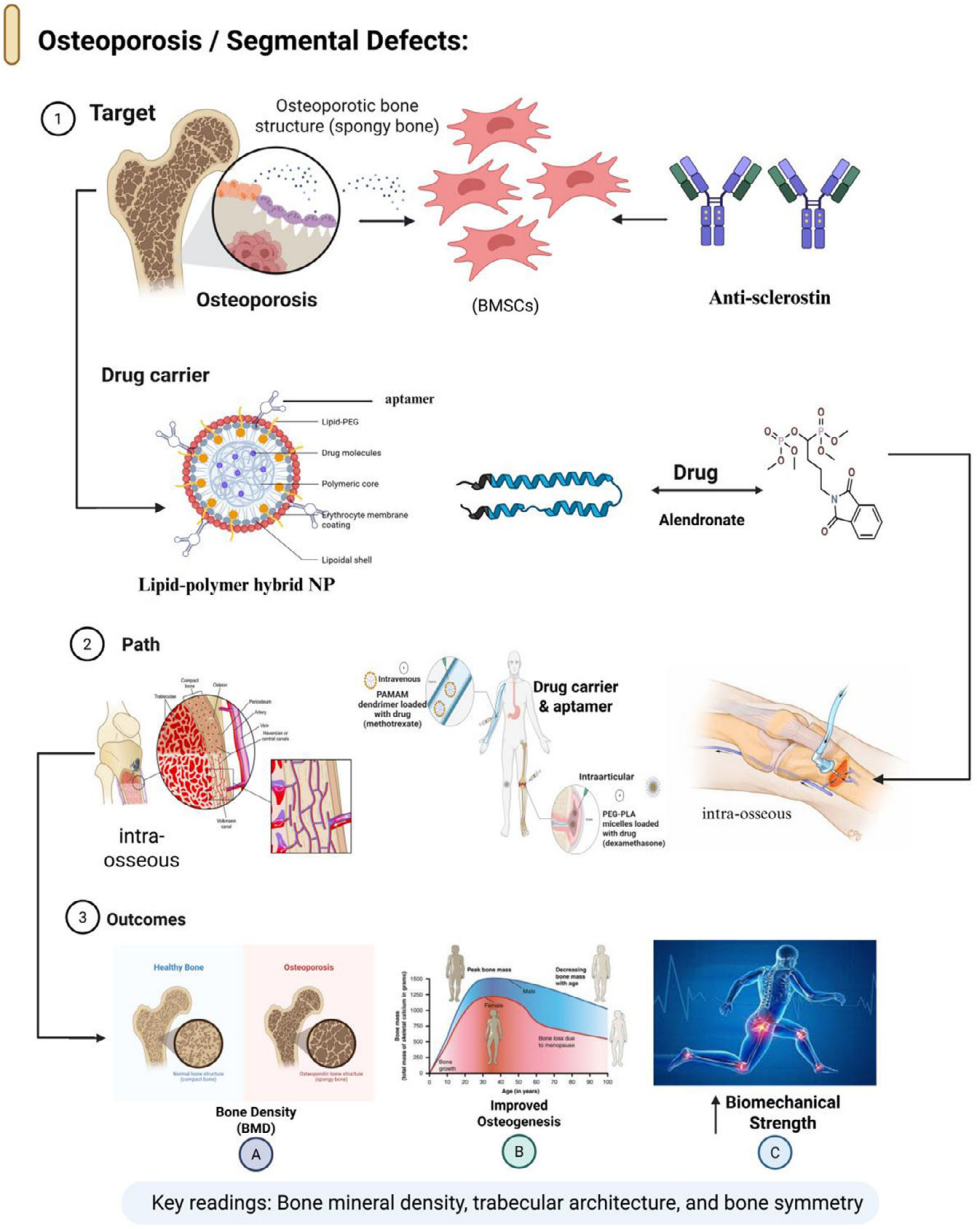
Aptamer‐enabled strategies for osteoporosis and segmental bone defects. Schematic representation of aptamer‐based approaches for bone regeneration in osteoporotic bones and segmental defects. Aptamers are integrated into injectable carriers or load‐bearing scaffolds to enhance the recruitment and retention of osteogenic cells, modulate the local inflammatory and anabolic microenvironments, and support the bridging of critical‐sized defects. Regeneration was evaluated using microcomputed tomography, BMD, trabecular and cortical architecture, and mechanical competence, enabling a direct comparison of aptamer‐functionalized constructs with conventional bone grafting or growth factor‐based strategies. Representative systems include CH6‐functionalized tetrahedral DNA nanostructures embedded in GelMA hydrogels for mandibular defect repair [50], Apt19S‐conjugated anisotropic scaffolds for meniscal regeneration [52], and bone‐homing aptamer‐decorated polymeric nanoparticles for skeletal lesion targeting [53]. This figure was created using BioRender.com.

The performance of aptamer‐functionalized constructs for musculoskeletal delivery is governed by three interdependent design parameters: binding affinity, ligand density (or valency), and spacer/linker architecture. These variables act as key engineering controls that determine whether a construct can effectively navigate and engage molecular targets within the bone, cartilage, synovium, and marrow (Figure 2).

The binding affinity, quantified by the dissociation constant, defines the probability of target engagement. In musculoskeletal tissues, aptamers must bind within crowded extracellular matrices containing abundant proteins and charged macromolecules, while penetrating dense cartilage or synovial barriers [70]. Nanomolar or sub‐nanomolar affinities are typically required to achieve effective on‐target occupancy without necessitating excessively high doses, especially when facing protein corona formation and biological transport limitations [71, 72]. Specificity is equally critical; in protein‐rich environments, nonspecific binding and electrostatic interactions with negatively charged cartilage can lead to misleading targeting results unless rigorous physiologic‐condition assays are performed [71, 73].

Ligand density and valency shape the avidity landscape of the construct. Higher aptamer loading on nanoparticle or scaffold surfaces can enhance multivalent interactions with osteoblast‐lineage cells, chondrocytes, synoviocytes, tumor cells in skeletal lesions, or pathogens in infected bones [72, 74]. However, excessive ligand density may result in steric hindrance, reduced flexibility, increased aggregation, or altered protein corona profiles, ultimately impairing targeting and accelerating clearance by macrophage‐rich organs [72, 75, 76]. This relationship is not linear; beyond a critical threshold, additional ligands degrade performance rather than improve it. Bone‐targeted dual‐ligand systems illustrate how surface stoichiometry influences biodistribution and reinforce the need to treat ligand density as a tunable, quantitative design parameter [74].

Spacer and linker chemistries further influence effective target binding. Direct conjugation of aptamers to nanoparticle cores can constrain flexibility and induce electrostatic interference, reducing apparent binding despite favorable affinity in solution‐phase assays. Flexible linkers, such as polyethylene glycol chains, aliphatic spacers, or nucleotide‐based extensions, can project the aptamer beyond protective coatings and restore access to buried or matrix‐embedded epitopes on osteoid surfaces or within cartilage and synovium [77, 78]. The spacer length and immobilization mode critically affect the binding geometry and kinetic readouts in surface‐based assays. For cartilage‐targeted constructs, reduced hydrodynamic size and compliant surfaces facilitate proteoglycan mesh penetration, whereas larger constructs with shorter linkers may be suitable for exposed mineralized targets [70].

A major limitation in current musculoskeletal aptamer studies is the underreporting of these engineered parameters. Claims of enhanced targeting or efficacy are often qualitative and lack details on dissociation constants, ligand density, or linker identity. To support reproducibility and design advancement, studies must provide affinity values measured under physiological conditions, report aptamer loading per surface area, specify linker composition and length, and disclose core particle features such as size distribution, polydispersity index, and zeta potential [72, 79].

### 2.4 Musculoskeletal Target Taxonomy: Cell‐, Matrix‐, and Pathway‐Directed Aptamers

Musculoskeletal disease aptamers can be organized into a pragmatic taxonomy based on whether they primarily target cells, extracellular matrix components, or disease‐relevant signaling pathways. This classification reflects how aptamers are deployed within nanocarriers and biomaterial systems and provides a functional framework for translational design [47].

Cell‐directed aptamers bind to defined cellular populations that drive bone and joint pathologies. The osteoblast‐binding aptamer CH6 was identified through selection against osteoblast‐like cells and retains low‐nanomolar affinity when incorporated into larger nucleic acid constructs [49]. When displayed on tetrahedral DNA nanostructures and integrated into GelMA hydrogels, CH6 enables selective recruitment and activation of osteoblast‐lineage cells in osteoporotic mandibular defects, resulting in improved bone regeneration and mechanical performance compared with nontargeted controls [49]. Similarly, the mesenchymal stromal cell‐targeting aptamer, Apt19S, has been used to recruit endogenous stromal cells. Its conjugation to anisotropic meniscal scaffolds enhanced in situ stromal cell recruitment and supported region‐specific fibrocartilage regeneration [51]. Apt19S has also been incorporated into aptamer‐functionalized tetrahedral framework nucleic acids to enable targeted dual microRNA delivery and promote endogenous articular cartilage repair in vivo [80]. Beyond bone and cartilage, in vivo selection strategies have produced skeletal muscle‐targeting internalizing aptamers, demonstrating that comparable approaches can be extended across musculoskeletal tissues when biological barriers and pharmacokinetic constraints are incorporated into selection pressure [54].

Matrix‐directed aptamers are designed to anchor delivery systems to structural components, such as hydroxyapatite and other mineral‐associated interfaces, thereby improving localization within mineralized or fibrocartilaginous tissues. Hydroxyapatite‐binding aptamers have been employed as targeting ligands for bone marrow stromal cells and bone‐associated delivery contexts, supporting prolonged localization at osteotomy sites or osteolytic lesions where mineral exposure is high [81, 82]. Such matrix binding can prolong local exposure by tethering payloads to relatively stable matrix components, even as cell populations remodel over time.

Pathway‐directed aptamers bind soluble mediators or pathway regulators that are implicated in the progression of musculoskeletal diseases. Sclerostin, a secreted inhibitor of Wnt signaling, is a key negative regulator of bone formation. Loop‐specific anti‐sclerostin aptamers have been developed to modulate sclerostin activity and promote bone‐anabolic responses in vivo, illustrating the feasibility of designs that combine pathway antagonism with targeting functionality [83, 84]. In inflammatory arthritis, aptamers targeting cytokines and profibrotic mediators offer a complementary strategy. An interleukin‐6 RNA aptamer has been evaluated in arthritis‐relevant settings [41], while a CTGF DNA aptamer suppressed pannus formation and joint inflammation in collagen‐induced arthritis, supporting both diagnostic and therapeutic potential in RA [85]. Synovial fibroblast‐targeting aptamers selected against RA‐relevant fibroblasts further demonstrate that disease‐context selection enables direct engagement of the pannus microenvironment [38].

Infection‐directed aptamers are increasingly associated with orthopedic complications. DNA aptamers and aptamer‐guided delivery strategies targeting *Staphylococcus* aureus and biofilm‐associated structures support a potential role in osteomyelitis and prosthetic joint infection when integrated with antibiotic depots or implant coatings [86, 87]. Collectively, this cell, matrix, and pathway taxonomy highlights how aptamer discovery and engineering variables, including selection strategy, stabilization chemistry, affinity optimization, valency tuning, and spacer design, can be systematically applied across osteogenesis, cartilage repair, synovial inflammation, tumor‐associated bone disease, and infection, with the target class determined by therapeutic intent [47].

### 2.5 Mini‐Summary: Key Aptamer Design Variables for Musculoskeletal use

**FIGURE 3:**
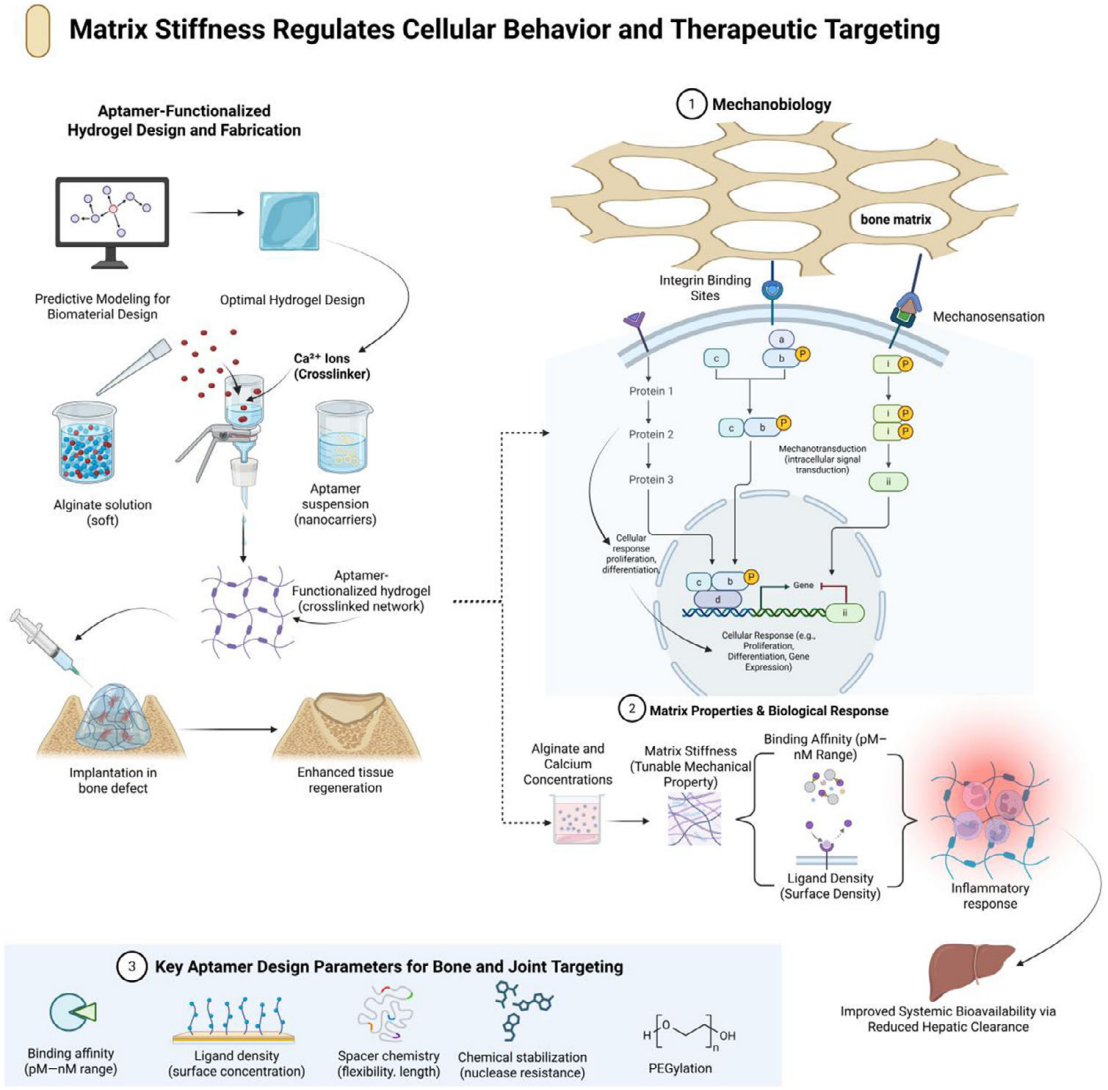
Key aptamer design variables for bone and joint targeting. Conceptual map of design parameters governing aptamer‐guided targeting in musculoskeletal tissues. Predictive modeling links matrix stiffness, tuned by alginate and calcium concentration, to cellular mechanosensation and mechanotransduction in the bone matrix, thereby influencing cell behavior and inflammatory responses. These mechanical cues are integrated with molecular design variables, binding affinity, ligand density, spacer chemistry, and chemical stabilization (for example, 2′‐fluoro, locked nucleic acid, and PEGylation) to determine in vivo biodistribution, off‐target accumulation (such as in the liver), and retention at bone and joint sites. Optimization across these axes yields constructs with maximized tissue regeneration and controlled systemic exposure to the drug. Design principles are informed by studies demonstrating the effects of ligand density and valency on nanoparticle biodistribution [72, 74], spacer chemistry on target accessibility [77, 90], and chemical stabilization strategies on aptamer half‐life in musculoskeletal microenvironments [59, 91, 92]. This figure was created using BioRender.com.

Across bone and joint applications, the success of aptamer‐functionalized nanocarriers is governed by a limited set of interdependent design variables. Effective competition within complex skeletal environments and avoidance of off‐target sequestration require ligands with high affinity and specificity, typically within the low‐nanomolar range [88, 89]. Chemical stabilization strategies, including two‐prime sugar modifications, partial locked nucleic acid incorporation, and selected backbone and stealth chemistries, are critical for preserving aptamer structure and binding capacity in plasma, bone marrow, and synovial fluid, all of which contain high nuclease activity [28, 39]. Ligand density and valency must be balanced within an intermediate range that enhances avidity without promoting aggregation or accelerated clearance by the RES [74]. Spacer and linker architectures must provide sufficient conformational flexibility and epitope accessibility to support binding within dense cartilage matrices or on mineralized bone surfaces [74].These principles are summarized in the musculoskeletal aptamer panel presented in Table 1,with the core design axes illustrated in Figure 3.

**TABLE 1:** Musculoskeletal aptamer panel: targets, chemistry, and typical uses.

Aptamer name	Primary target	**Indication** **(s) used or proposed**	Nucleic acid type & affinity	Key chemical features	Representative carrier contexts
CH6	Osteoblast‐lineage cellsReceptor: Osteoblast surface epitope (cell‐SELEX; identity undetermined)	Osteoporotic mandibular defect; broader bone regenerationValidation: In vivo OVX rat; ↑ bone volume, ↑ trabecular connectivity, improved defect bridging	DNA aptamer; low‐nM affinity for osteoblast‐like cells (cell‐SELEX)	Used within TDNs; can incorporate 2′ modifications for stability; often combined with hydrogel depots	TDNs embedded in GelMA hydrogels for defect repair; conceptual use on systemic NPs for bone targeting (Hong et al., 2024; He et al., 2025)
Apt19S	MSCs/BMSCsReceptor: BMSC surface epitope (cell‐SELEX; identity undetermined)	Meniscal regeneration; bone/cartilage defect repairValidation: In vivo rabbit; ↑ MSC homing, ↑ fibrocartilage matrix, improved meniscal filling	DNA aptamer; low‐nM affinity for MSCs (cell‐SELEX)	Typically immobilized on solid scaffolds; compatible with additional stabilization (such as phosphoramidate linkages)	Surface‐conjugated on anisotropic collagen scaffolds and DNA hydrogels to recruit endogenous MSCs to meniscal and bone defects (Li et al., 2022)
Bone‐homing aptamer (Chen et al., 2019)	Bone metastasis microenvironment/mineralized bone nicheReceptor: Hydroxyapatite/mineralized matrix‐associated epitope	Bone‐metastatic prostate cancer; conceptual extension to osteoporosis and osteomyelitisValidation: In vivo mouse; ↑ skeletal accumulation, ↑ bone:RES ratio, ↓ off‐target signal	DNA aptamer selected by in vivo SELEX; high relative affinity for bone vs soft tissues	Nuclease‐resistant chemistry; ligand density optimized on NP surfaces; often PEGylated backbone	Conjugated to polymer/lipid NPs to enhance accumulation in bone lesions and improve bone:RES biodistribution indices (Chen et al., 2019)
Chondrocyte/cartilage‐matrix aptamers	Chondrocytes or type II collagen‐rich cartilage matrixReceptor: Type II collagen epitope or chondrocyte surface marker	OA cartilage repair and focal lesionsValidation: In vivo rodent OA; ↓ OARSI scores, ↑ proteoglycan, ↑ type II collagen retention	DNA/RNA aptamers; typically low‐nM affinities (cell‐SELEX)	Frequently combined with PEG linkers; may include 2′‐O‐methyl or 2′‐F substitutions for stability in synovial fluid	Decorated on sub‐100 nm liposomes or polymer NPs for intra‐articular delivery of miRNAs/anti‐catabolic agents (Ji et al., 2021; He et al., 2025)
IL‐6 aptamer	Interleukin‐6 cytokineReceptor: IL‐6 cytokine (soluble; direct binding)	Collagen‐induced arthritis; conceptual use in RA and spondyloarthritisValidation: In vitro/preclinical arthritis; IL‐6 neutralization, ↓ inflammatory signaling	DNA aptamer with high affinity for IL‐6	Stabilized with 2′ modifications and phosphorothioate linkages in some studies; designed for systemic or local administration	Administered as free aptamer or simple carrier formulations; natural candidate as payload or ligand on RA‐targeted depots and NPs (Nishina et al., 2003; Shatunova et al., 2021)
CTGF aptamers	CTGF (overexpressed in RA FLS)Receptor: CTGF (soluble; direct binding)	Rheumatoid arthritisValidation: In vivo CIA model; ↓ pannus formation, ↓ joint inflammation	DNA/RNA aptamers with high affinity for CTGF (study‐dependent)	Chemically modified for nuclease resistance and reduced off‐target binding; may use PEG or lipid conjugation	Used as free agents or integrated into local delivery systems targeting RA synovium; candidate ligands for synovial‐homing nanocarriers (Wu et al., 2022; Shatunova et al., 2021)
Sclerostin aptamers	Sclerostin (osteocyte‐derived Wnt antagonist)Receptor: Sclerostin loop domains (Wnt antagonist)	Osteoporosis; bone defect repairValidation: In vivo; bone‐anabolic response, ↑ osteoblast activity, ↑ bone formation markers	RNA or DNA aptamers with nanomolar affinity	Developed with 2′‐F/LNA substitutions; sometimes PEGylated to extend half‐life	Used as antagonistic ligands and analytical tools; suitable as dual‐function components (targeting + payload) on bone‐targeted nanocarriers (Lee et al., 2021)
Bacterial aptamers (such as anti–S. aureus)	Pathogen surface antigens/biofilm components Receptor: S. aureus protein A or biofilm surface structures	Systemic and local bacterial infections; conceptual extension to osteomyelitis and prosthetic joint infectionValidation: Primarily in vitro; bacterial detection and capture; no in vivo MSK data yet	DNA/RNA aptamers; affinity varies by strain and antigen	Typically modified for nuclease resistance; often include fluorophores or drug‐conjugation handles	Decorated on gold or polymer NPs for targeted antimicrobial delivery, biofilm disruption, and theranostic imaging; adaptable to bone infection depots and implant coatings (Ye et al., 2024)

Future progress in the field will depend on rigorous quantitative reporting of these variables and explicit linkages between design parameters, biodistribution behavior, and disease‐relevant outcomes. Establishing such relationships will enable the formulation of robust and generalizable design rules applicable across musculoskeletal indications and delivery platforms.

The design parameters outlined above—aptamer affinity, chemical stabilization, ligand density, valency, and spacer architecture—do not apply uniformly but must be tailored to the distinct biological and anatomical constraints of each musculoskeletal indication. The following sections examine how these variables are configured across osteoporosis, cartilage repair, inflammatory arthritis, bone tumors, and infection to achieve indication‐specific targeting and therapeutic outcomes.

## 3 Indication Landscape and Target–Payload Matchmaking

Aptamer‐functionalized nanocarriers for musculoskeletal diseases can be organized along three overlapping axes: indication, target class (cell, matrix, or pathway), and payload. In osteoporosis and segmental defects, cartilage and meniscus pathology, inflammatory arthritides, infection/osteomyelitis, bone tumors, and metastases, the most attractive systems pair (i) a biologically validated target, (ii) a low‐nanomolar, chemically stabilized aptamer, and (iii) a carrier that is tuned to the anatomical requirements of bone, cartilage, synovium, or marrow. Table 2 summarizes the principal in vivo and ex vivo platforms, whereas Table 1 presents the aptamer panel.

**TABLE 2:** Aptamer‐functionalized nanocarriers across bone and joint indications.

Indication / model	Target & aptamer (type)	Carrier / formulation	Route	Payload / function	Key particle parameters (if reported)	Main outcomes vs control	Key references
Osteoporosis / mandibular defect (OVX rat)Model: In vivo, OVX rat mandibular defect	Osteoblasts – CH6 DNA aptamer (cell‐SELEX, low‐nM affinity)Molecular target: Osteoblast surface epitope; receptor identity undetermined	CH6‐decorated tetrahedral DNA nanostructures embedded in GelMA hydrogel	Local defect filling	Osteogenic stimulation and bone regeneration via targeted recruitment of osteoblast‐lineage cells	TDN size in tens of nm; GelMA pore scale in µm range; aptamer density optimized qualitatively on TDN surface	↑ Bone volume fraction, ↑ trabecular connectivity, improved defect bridging and biomechanical strength vs non‐targeted GelMA or TDN	Hong et al., 2024; He et al., 2025
Osteoporosis / bone metastasis (mouse)Model: In vivo, mouse bone metastasis	Bone‐homing DNA aptamer (in vivo SELEX; enriched in skeletal lesions)Molecular target: Bone mineral/hydroxyapatite‐associated epitope	Aptamer‐decorated polymeric nanoparticles	Intravenous	Chemotherapeutic or siRNA delivery to bone lesions	NP size ∼100–150 nm; mildly negative zeta potential; ligand density reported qualitatively	↑ Accumulation in bone metastases, ↑ bone:RES uptake ratio, ↓ liver/spleen off‐target signal; improved lesion control vs non‐aptamer NP	Chen et al., 2019; He et al., 2025
Segmental / meniscal defect (rabbit or rat)Model: In vivo, rabbit/rat meniscal defect	MSC‐recruiting aptamer – Apt19S (cell‐SELEX, low‐nM affinity for BMSCs)Molecular target: BMSC surface receptor; identity undetermined	Apt19S‐functionalized anisotropic scaffolds (such as 3D collagen/polymer)	Local implantation	Endogenous MSC recruitment and anisotropic meniscal regeneration	Scaffold pores ∼100–300 µm; anisotropic architecture characterized; aptamer immobilized on surface	↑ MSC homing, ↑ fibrocartilage‐like matrix, better defect filling and biomechanical performance vs non‐targeted scaffold	Li et al., 2022; He et al., 2025
OA cartilage (rodent models)Model: In vivo, rodent OA cartilage	Chondrocyte‐ or cartilage‐matrix–targeting aptamers (cell‐SELEX against chondrocytes/type II collagen‐rich matrix)Molecular target: Chondrocyte surface marker or type II collagen epitope	Aptamer‐decorated nanoparticles or hydrogels	Intra‐articular	Delivery of miRNA/siRNA or anti‐catabolic small molecules into cartilage	Particle size typically <100 nm for cartilage penetration; PEGylated flexible linkers as spacers; moderately negative zeta potential	↓ OARSI histological scores, ↑ proteoglycan content, ↑ indentation stiffness vs untargeted carriers	Ji et al., 2021; He et al., 2025
RA inflammatory arthritis (CIA and related models)*Model: In vivo, CIA mouse/rat	Synovial fibroblasts/macrophages and cytokines (such as IL‐6, CTGF) – DNA aptamersMolecular target: IL‐6 cytokine; CTGF; RA‐FLS surface marker	Aptamer‐conjugated DNA nanostructures, liposomes, or hydrogels (emerging)	Intra‐articular or intravenous	Neutralization of IL‐6 or CTGF; targeted delivery of anti‐inflammatory agents or siRNA to inflamed synovium	Typical NP size ∼50–150 nm in conceptual and early preclinical systems; ligand density moderate; zeta potential slightly negative	↓ Arthritis scores, ↓ synovial cytokines, ↓ pannus formation, ↑ joint preservation vs non‐targeted formulations in proof‐of‐concept aptamer studies	Nishina et al., 2003; Wu et al., 2022; Shatunova et al., 2021; Hu et al., 2024
Osteosarcoma (orthotopic/subcutaneous models)Model: In vivo, orthotopic/subcutaneous tumor mouse	Tumor‐associated receptors (such as nucleolin, CSC markers such as CD133, EpCAM) – DNA/RNA aptamersMolecular target: Nucleolin (AS1411); CD133; EpCAM	Aptamer‐decorated liposomes, polymeric NPs, micelles	Intravenous	Targeted delivery of cytotoxics (such as doxorubicin) and/or imaging agents	Sizes typically 80–150 nm; PEGylated surface; aptamer valency optimized to avoid aggregation	↓ Tumor volume, ↓ metastatic spread, ↑ survival; in some models, preserved bone integrity versus nontargeted chemotherapy	Shigdar et al., 2021; Zhu & Chen, 2018; Mohammadinejad et al., 2024
Bone metastasis (prostate/breast cancer)Model: In vivo, mouse bone metastasis	Bone‐targeting aptamers from in vivo SELEX (bone‐lesion–preferential)Molecular target: Bone‐lesion‐enriched epitope (in vivo SELEX)	Aptamer‐decorated NPs or liposomes	Intravenous	Chemotherapy, radionuclides, or siRNA targeted to skeletal metastases	NP size ∼100–150 nm; bone‐homing aptamers on surface; ligand density optimized experimentally	↑ Bone localization, ↓ off‐target liver/spleen accumulation, improved control of bone lesions versus nontargeted NP	Chen et al., 2019; Benner et al., 2020; Mohammadinejad et al., 2024
Osteomyelitis/bone infection (conceptual stage)Model: Conceptual/in vitro only	Bacterial surface antigens/biofilm components – anti–Staphylococcus aureus and related aptamersMolecular target: S. aureus protein A or biofilm‐associated antigen	Conceptual: aptamer‐functionalized antibiotic‐loaded NPs, hydrogels, or implant coatings	Local (defect or implant coating) or systemic	Pathogen‐specific capture, antimicrobial delivery, and biofilm disruption in bone and around prostheses	Particle parameters not yet reported for bone‐specific models; designs extrapolated from nonbone infection studies	No dedicated preclinical osteomyelitis data yet; strong theoretical fit for local depots and prosthetic joint infection management	Ye et al., 2024; Shatunova et al., 2021; Zhu & Chen, 2018

*Note:* This table summarizes the major aptamer–nanocarrier configurations evaluated or proposed in musculoskeletal models, organized by indication. The particle parameters are reported as described in the original studies. To date, most RA entries reflect ligand‐level or early delivery work; fully mature RA, specific aptamer–nanocarrier systems are still emerging.

### 3.1 Osteoporosis and Bone Regeneration/Segmental Defects

The primary engineering challenge in osteoporosis and segmental bone defects is to convert diffuse osteoinductive cues into localized, mechanically competent bone formation in poorly vascularized, mechanically stressed environments. Aptamer‐functionalized systems, which direct bone‐forming or progenitor cell targets or depots to the mineralized matrix, overcome this limitation.

CH6 is an osteoblast‐binding aptamer with cell specificity. CH6 was identified using cell‐SELEX against osteoblast‐like cells, optimized to low‐nanomolar affinity, incorporated into tetrahedral DNA nanostructures (TDNs), and embedded within GelMA hydrogels to repair localized defects in osteoporotic mandibles [49]. To compare transplantation research in an ovariectomized rat model with CH6‐TDN‐GelMA constructs, improved recruitment and differentiation of osteoblast‐lineage cells with increased bone volume fraction, enhanced trabecular connectivity, and biomechanical strength were observed [49]. This strategy establishes an overall therapeutic approach for osteoporotic defects: combining an osteoblast/MSC‐targeting aptamer with a slowly degradable depot that can withstand local inflammatory and mechanical pressures (Figure 3).

Another complementary method is the use of matrix‐binding or bone‐homing aptamers to target carriers to mineralized surfaces or osteolytic niches. Chen and colleagues (2019) applied in vivo SELEX in a bone metastasis model of prostate cancer to discover a bone‐homing DNA aptamer that selectively accumulates in skeletal lesions and reduces liver and spleen accumulation of untargeted backbones when conjugated to nanoparticles. Designed to treat cancer, the selection scheme inherently selects ligands with good bone: RES biodistribution, which is directly useful in the treatment of systemic osteoporosis or metastatic bone diseases [53].

The third method involves sclerostin‐directed aptamers. Sclerostin, an osteocyte‐secreted Wnt antagonist, is a potent inhibitor of bone formation. Monoclonal antibodies can neutralize sclerostin, thereby increasing bone mass and microarchitecture in patients with high‐risk osteoporosis [93]. Nanomolar sclerostin‐binding aptamers have been developed to detect and inhibit sclerostin [94]. These ligands may be presented on nanoparticles or scaffolds to combine two advantages: activated delivery of the Wnt pathway and targeted delivery to osteocyte‐rich regions, serving as delivery agents and targeting molecules (Table 1).

In these systems, microcomputed tomography (uCT) and BMD are the most common structural measures, and histology and mechanical testing are the most common functional repair outcome measures. In general, the new data indicate that aptamer‐based depot engineering can be a dependable approach to target osteogenesis at specific defects or osteoporotic locations, instead of utilizing nonspecific scaffold placement or systemic osteoanabolic cues [47, 49, 53, 94].

### 3.2 Cartilage and Meniscus

Cartilage and meniscus repair face various challenges, including avascular tissue, dense extracellular matrix, and mechanical stress. In this respect, optimal aptamer designs should be directed towards the selective recruitment of reparative cells and their long‐term retention in cartilage or fibrocartilage, rather than the general homing of the entire system.

One of the most notable effects is the recruitment of MSCs by Apt19S. Li et al. developed an anisotropic collagen biomimetic scaffold to repair meniscal cartilage and surface‐coated it with Apt19S, an aptamer with low‐nanomolar specificity for BMSCs obtained through cell‐SELEX [43]. Apt19S‐conjugated scaffolds enhanced in situ MSC homing and fibrocartilage‐like matrix deposition and restored meniscal shape and mechanical functions more effectively than nontargeted scaffolds in a small‐animal model of meniscal defects [43, 47]. This study demonstrates that a passive scaffold can be converted into an active recruiter of native progenitors using one aptamer–aptamer affinity and a well‐designed aptamer (Tables 1 and 2).

**FIGURE 4:**
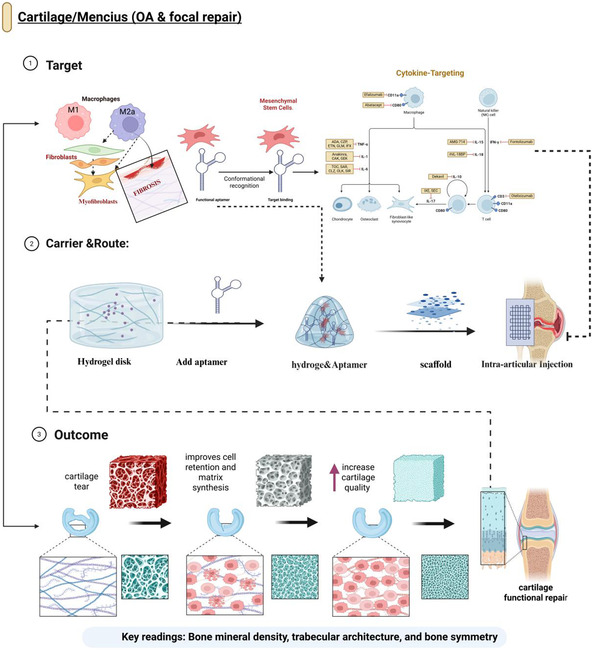
Aptamer‐functionalized hydrogels for cartilage and meniscus repair in osteoarthritis and focal defects. Conceptual framework for aptamer‐guided regeneration of cartilage and the meniscus. The “Target” tier focuses on the fibrotic and inflamed joint microenvironment, including M1/M2a macrophages, fibroblasts/myofibroblasts, mesenchymal stem cells, and pathogenic cytokine networks that drive cartilage degeneration, which are recognized by disease‐relevant aptamers. In the “Carrier and route” tier, aptamers are incorporated into a hydrogel disk, forming an aptamer‐functionalized hydrogel or scaffold that is delivered via intraarticular injection and local implantation, enabling spatially confined, sustained, and cell‐responsive binding within the joint. The “Outcome” tier illustrates improved cell retention, matrix synthesis, and cartilage quality, leading to structural repair of cartilage tears and restoration of joint function, with key readouts including cartilage morphology, subchondral BMD, trabecular architecture, and joint symmetry. Relevant studies include aptamer‐functionalized tetrahedral framework nucleic acids for dual microRNA delivery in articular cartilage repair [95], Apt19S‐conjugated scaffolds for region‐specific meniscal fibrocartilage regeneration [49], and chondrocyte‐targeting aptamers for proteoglycan retention in osteoarthritis models. This figure was created using BioRender.com.

Based on this concept, Chen et al. (2023) developed an aptamer‐mediated strategy to recruit endogenous synovial‐derived reparative cells for meniscal healing in a rat model. They engineered bispecific synovial–meniscal aptamers, in which one aptamer recognized synovial cell populations and the other bound meniscal tissue, enabling cell capture and retention at the lesion site without exogenous cell transplantation. In an avascular meniscal injury setting, the bispecific design increased synovial cell accumulation within the defect, promoted fibrocartilaginous matrix deposition, and improved mechanical properties compared with monospecific aptamer controls or nonaptamer scaffolds [64]. Conceptually, this represents a higher‐order targeting paradigm in which aptamer ‘tethers’ connect an endogenous progenitor‐rich niche to the injury microenvironment, effectively converting local biology into a regenerative depot (Figure 4).

Direct attacks can also be performed on cartilage. In osteoarthritis (OA) models, cell‐SELEX identified chondrocyte‐ and cartilage matrix‐binding aptamers against either primary chondrocytes or type II collagen‐rich matrices conjugated to liposomes and polymer nanoparticles [47, 96]. Following the dense proteoglycan network, these constructs are normally entrusted with sub‐100 nm‐sized particles, as well as a flexible PEG linker, to enhance intraartilage retention and delivery of miRNAs or anti‐catabolic agents, ultimately decreasing OARSI histological scores and maintaining proteoglycan stores [96].

Cartilage and meniscus applications have resulted in outcomes such as OARSI scores, proteoglycan staining, indentation/compressive modulus, and imaging defect filling and integration. Together, aptamers in the form of Apt19S‐based scaffolds, bispecific synovial‐meniscal constructs, and chondrocyte‐directed nanocarriers (Tables 1 and 2) demonstrate that aptamers can not only be utilized to deliver a hit to the cartilage but also to correctly guide reparative cells into the right microanatomical niches at the right time.

### 3.3 Rheumatoid Arthritis and Spondyloarthritis

**FIGURE 5:**
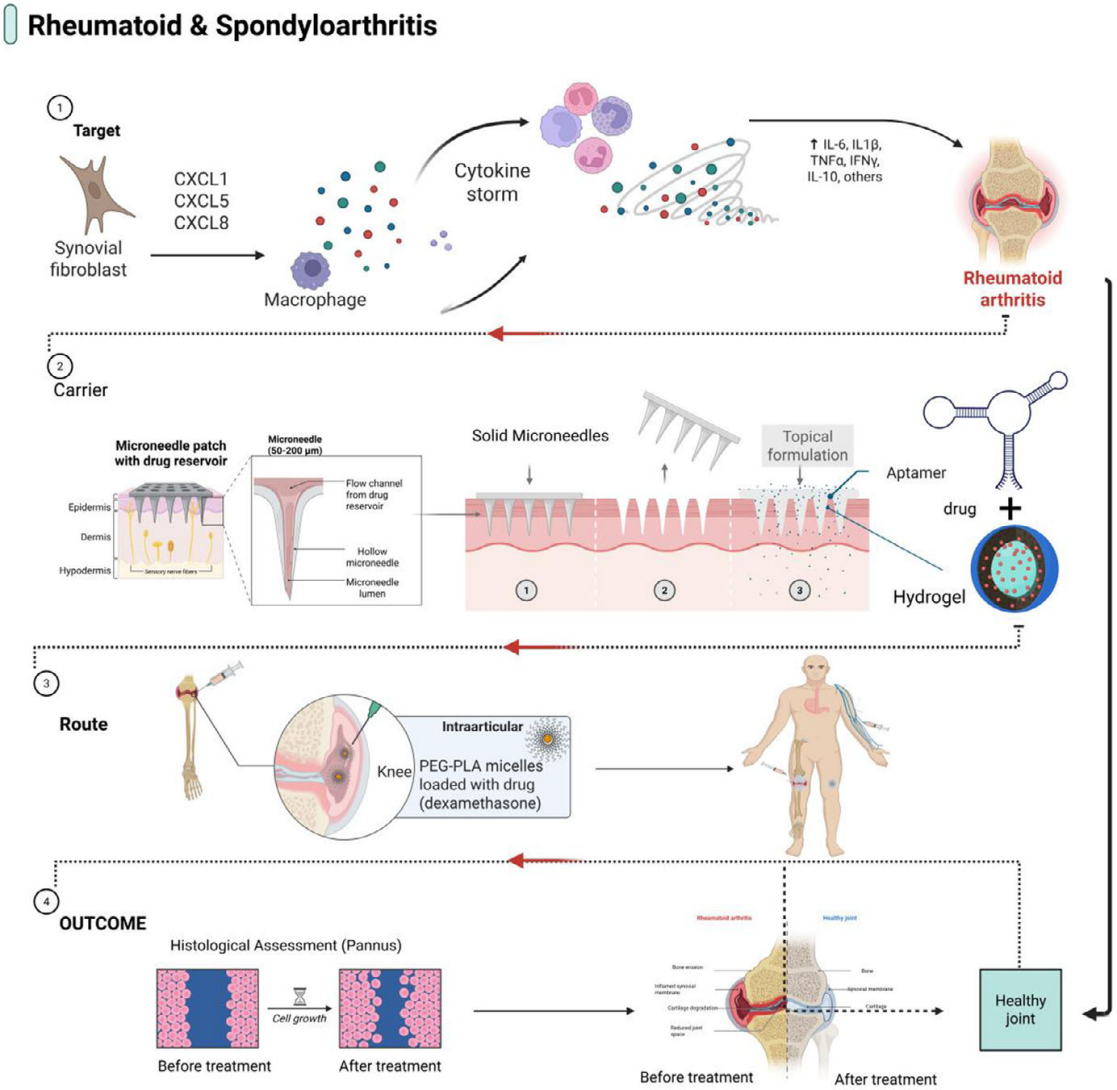
Aptamer‐based strategies for RA and spondyloarthritis. Illustration of aptamer‐guided interventions targeting synovial inflammation in RA and spondyloarthritis. Aptamers are designed to recognize inflamed synovial tissue, activated macrophages, FLs, key cytokines, and costimulatory molecules. These ligands are displayed on nanoparticles, hydrogels, or biological carriers to enable the selective delivery of anti‐inflammatory or disease‐modifying cargo via intraarticular or systemic routes. The expected outcomes include reduced synovial inflammation, attenuation of structural damage, and improved joint function, evaluated using clinical scores, imaging endpoints, and histological analyses. Key examples include synovial fibroblast‐targeting aptamers selected against RA‐relevant fibroblasts, an IL‐6 RNA aptamer evaluated in arthritis‐relevant settings [41], and a CTGF DNA aptamer that suppressed pannus formation and joint inflammation in collagen‐induced arthritis [85]. This figure was created using BioRender.com.

In RA and related spondyloarthritides, the primary therapeutic focus shifts from structural cells to the inflamed synovium, particularly fibroblast‐like synoviocytes (FLS) and synovial macrophages, which support pannus formation, cytokine secretion and joint damage [6]. Aptamer‐based approaches in this field target pathway‐specific ligands and synovial‐homing sequences that can be attached to intraarticular depots, microneedle devices, or small stealthy nanoparticles, as illustrated in Figure 5.

Evidence from early studies targeting proinflammatory cytokines supports the use of pathway‐specific aptamers in this context. In collagen‐induced arthritis, DNA aptamers selected against interleukin‐6 (IL‐6) reduced joint swelling and structural damage by neutralizing IL‐6 signaling, demonstrating that high‐affinity nucleic acid ligands can adjust key cytokine pathways in vivo (Nishina et al., 2003). Recently, CTGF‐binding aptamers have been shown to lessen the aggressive behavior of RA FLS and decrease synovial hyperplasia in preclinical models, highlighting CTGF as an important synovial target [85]. Overall, IL‐6 and CTGF aptamers comprise the core of a synovial‐focused aptamer panel with strong translational potential (Table 1).

At the delivery level, RA offers an opportunity to use locally administered depot formulations via microneedles to increase drug exposure in periarticular tissues while limiting the systemic burden. Aptamer‐enabled microneedle platforms have been reported in arthritis models, supporting the feasibility of combining local depot delivery with nucleic‐acid therapeutics [97, 98]. Building on this, incorporating fibroblast‐like synoviocyte or cytokine‐targeting aptamers into microneedle tips, nanoparticle cargos, or hydrogel matrices represents a plausible route to further concentrating therapy within the pannus and mitigating cartilage and bone damage, although this integration remains largely developmental and requires systematic validation [24, 41].

Outcomes in RA and spondyloarthritis models include clinical arthritis scores, joint swelling, cytokine profiling, and histological assessment of the pannus and bone erosion. Currently, very few fully integrated aptamer–nanocarrier systems have been tested in vivo for RA, and the field remains largely at the stage of ligand discovery and platform prototyping (Table 2). From a target–payload matching perspective, however, the approach is simple: pair synovial‐homing or cytokine‐targeted aptamers with intraarticular depots, microneedles, or small PEGylated nanoparticles designed to target the inflamed synovium without rapid RES clearance (Figure 5).

### 3.4 Infection and Osteomyelitis

**FIGURE 6:**
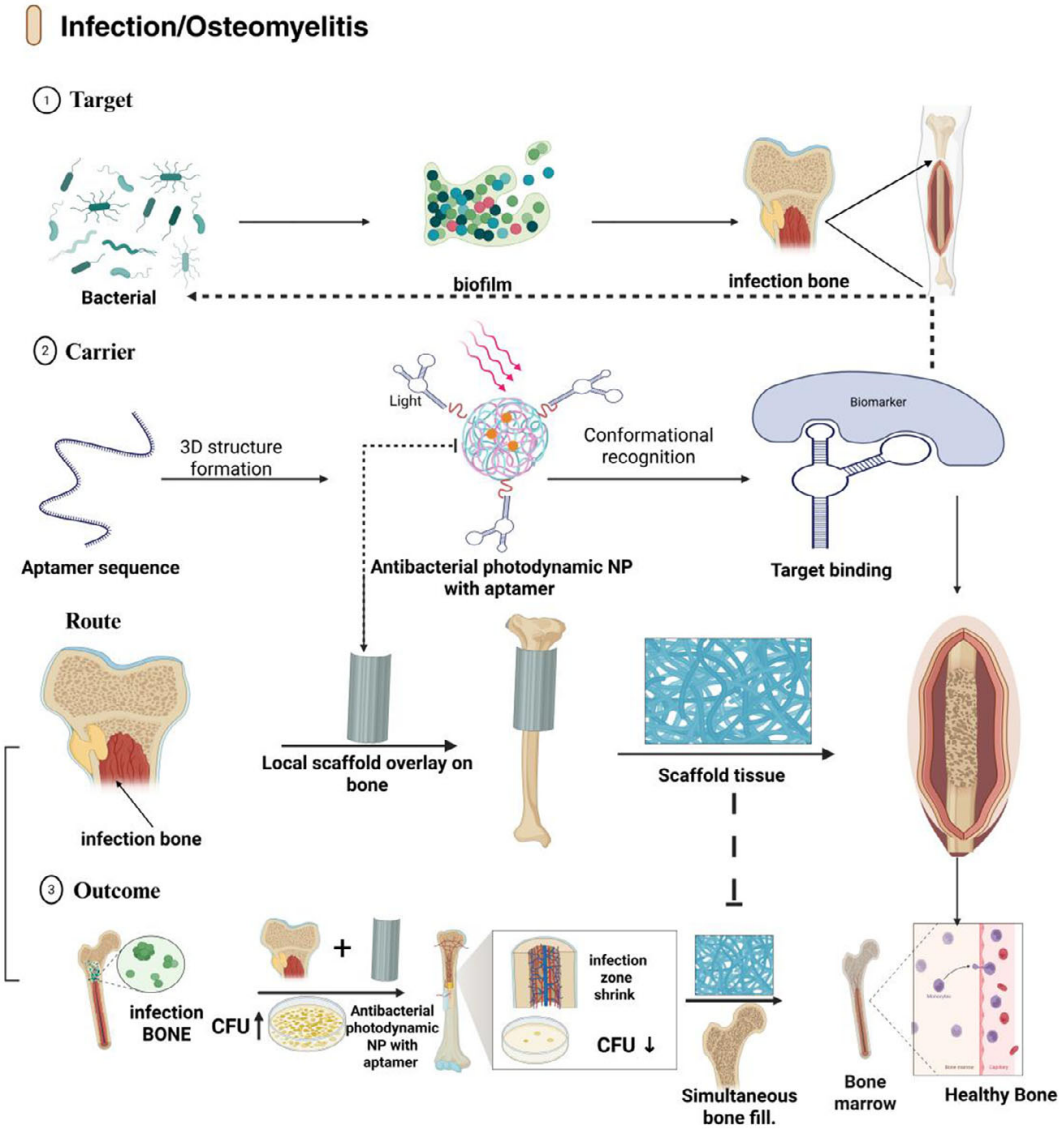
Aptamer‐guided platforms for treating bone infection and osteomyelitis. Conceptual overview of aptamer‐based strategies to eradicate bone infections and osteomyelitis. The targeting tier depicts bacterial populations and biofilms within the infected bone, as well as disease‐specific biomarkers that can be recognized by antibacterial aptamers. The carrier tier includes antibacterial photodynamic nanoparticles or other nanocarriers decorated with aptamers that undergo conformational recognition of bacterial or biofilm targets. The route tier illustrates the local application of aptamer‐functionalized scaffolds or coatings at the infected bone surface to achieve high local drug concentrations with limited systemic exposure. The outcome tier highlights the reduction in bacterial colony‐forming units, shrinkage of the infection zone, simultaneous filling of bone defects by scaffold‐supported regeneration, and eventual restoration of healthy bone marrow and cortical integrity. Aptamer‐guided strategies include anti‐Staphylococcus aureus DNA aptamers and aptamer‐targeted drug delivery systems for biofilm‐associated infections [87], with conceptual applications in prosthetic joint infection management. This figure was created using BioRender.com.

Bone and joint infections, especially chronic osteomyelitis and prosthetic joint infections, present distinct biophysical and biological challenges, as bacteria in biofilm‐coated bone and hardware are resistant to antibiotics and the host immune system. Aptamer‐functionalized systems have two key advantages in this respect: pathogen specificity and improved biofilm penetration, as shown in Figure 6.

A growing body of literature has reported aptamers targeting key bacterial pathogens (such as Staphylococcus aureus, Pseudomonas aeruginosa, and Escherichia coli). They have been incorporated into gold and polymer nanoparticles, hydrogels, and diagnostic platforms [87]. Nanoparticles functionalized with antibacterial aptamers have been shown to have better bacterial binding, antibiotic or photodynamic/photothermal delivery efficacy, and biofilm destruction than their nonfunctional counterparts in nonbone infection models [28, 87]. These reports share a common design philosophy: antibacterial aptamers can convert nonselective antimicrobial nanocarriers into pathogen‐targeted theranostics (Table 1).

The implementation of these designs for osteomyelitis will involve changes in carrier size, charge, and surface chemistry to reach the bone marrow and cortical bone and ensure that aptamer–bacterial interactions remain effective in pus‐ and protein‐rich microenvironments. To date, there are no bone‐specific applications, and Table 2 lists osteomyelitis and prosthetic joint infections as conceptual manifestations. Some of the proposed configurations include aptamer‐functionalized antibiotic‐loaded nanoparticles against S. aureus biofilms on the bone, aptamer‐hydrogel depots to release aptamers locally in infected defects, and surfaces on implants that use a combination of bacterial capture and local antimicrobial delivery [87].

Infection models have outcome measures, such as bacterial counts, biofilm biomass, inflammatory markers, and bone integrity, which can be assessed using imaging and histology. Osteomyelitis, infection, and prosthetic joint infection are frontier areas in which further development of musculoskeletal‐appropriate aptamer‐nanocarrier systems is needed, given the limited available information on these conditions. Nevertheless, the match between pathogen‐specific aptamers, local depots or coatings, and the anatomical characteristics of the infected bone is promising [28, 87].

### 3.5 Bone Tumors and Metastasis

**FIGURE 7:**
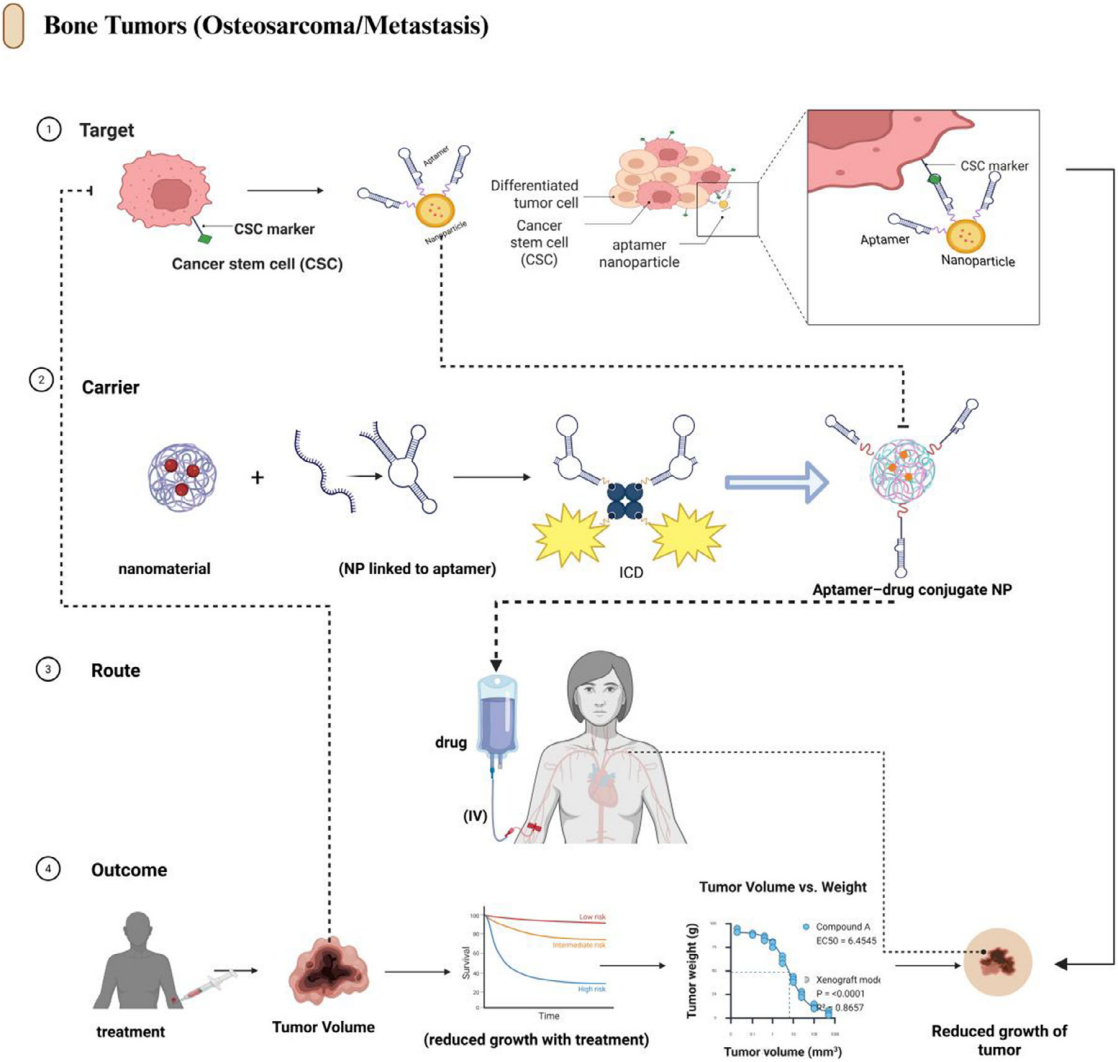
Aptamer‐guided nanotherapeutics for bone tumors and skeletal metastases. Therapeutic pathway for targeting primary bone tumors and skeletal metastases using aptamer‐decorated nanocarriers. The “Target” tier illustrates the identification of cancer stem cell markers and additional tumor‐associated antigens for selective aptamer recognition at the cellular level. The “Carrier engineering” tier shows the conjugation of tumor‐specific aptamers to nanoparticles or supramolecular constructs, enabling the multivalent binding and loading of cytotoxic or signaling cargo. The “Systemic administration” tier depicts the intravenous infusion of aptamer–nanoparticle formulations that preferentially accumulate in bone tumors and metastatic lesions while sparing healthy tissues. The “Outcome” tier highlights enhanced tumor cell killing, reduced tumor volume and weight, and potential eradication of micrometastatic disease, as reflected by the preclinical survival curves and tumor growth kinetics. Examples include nucleolin‐targeting aptamer AS1411‐functionalized nanocarriers for osteosarcoma, bone‐homing aptamers from in vivo SELEX for prostate cancer bone metastasis targeting [53], and aptamer‐guided photodynamic and photothermal therapeutic platforms for skeletal malignancies. This figure was created using BioRender.com.

The most developed applications of aptamer‐based targeting of the musculoskeletal system are currently bone tumors and skeletal metastases, which take advantage of decades of oncology‐related aptamer investigations and a broad range of bone‐targeting chemistries [99]. Aptamer–nanocarrier systems in this area engage cancer stem cell (CSC) markers, overexpressed tumor receptors, and bone‐homing sequences to selectively deliver cytotoxic or immunogenic cell death (ICD) payloads to cancerous tissues rather than normal bone tissues (Figure 7).

The most developed applications of aptamer‐based targeting in the musculoskeletal field are currently bone tumors and skeletal metastases, leveraging a strong oncology foundation in aptamer engineering, together with well‐established bone‐targeting delivery chemistries [21, 47, 99, 100]. In this domain, aptamer‐functionalized nanocarriers commonly engage cancer stem cell markers, overexpressed tumor receptors, and bone‐homing or bone‐associated targeting motifs to deliver cytotoxic therapeutics or ICD‐inducing payloads preferentially to malignant skeletal lesions while minimizing exposure to healthy bone [95, 101, 102, 103] (Figure 7).

The second method involves the use of bone‐homing aptamers identified by in vivo SELEX, as described by Chen et al. (2018), to form tumor‐ or CSC‐specific aptamer‐fusion proteins. These are thought to accumulate selectively in the bone first, then selectively bind tumor cells or CSCs, and optimize the bone: residue ratios and intralesional delivery of payloads [53]. Initial data indicate that dual‐targeted carriers have a higher level of tumor suppression and reduced off‐target toxicities than single‐ligand systems [99].

Finally, a good location for ICD‐inducing/theranostic payloads is bone tumors. Environment‐responsive release can be designed using aptamer‐coated nanoparticles to deliver ICD‐activating chemotherapeutics or photosensitizers, which have the potential to enhance the synergy with immune checkpoint inhibitors or adoptive cell therapies [29, 99]. Theranostic platforms, in which aptamers direct imaging agents and treatments to bone lesions, permit the detection and monitoring of responses and treatment within a single platform [43].

In general, the literature on bone tumors and metastases provides the most significant evidence that aptamer–nanocarrier complexes can be used to regulate biodistribution, increase efficacy, and enhance safety in musculoskeletal oncology (Table 2). Notably, they also provide a useful array of ligands and design concepts, including CSC markers, bone‐homing sequences, ICD payloads, and theranostics, which can be applied to nonmalignant bone and joint diseases.

## 4 Aptamer Nanocarrier Architectures and Conjugation Strategies

**FIGURE 8:**
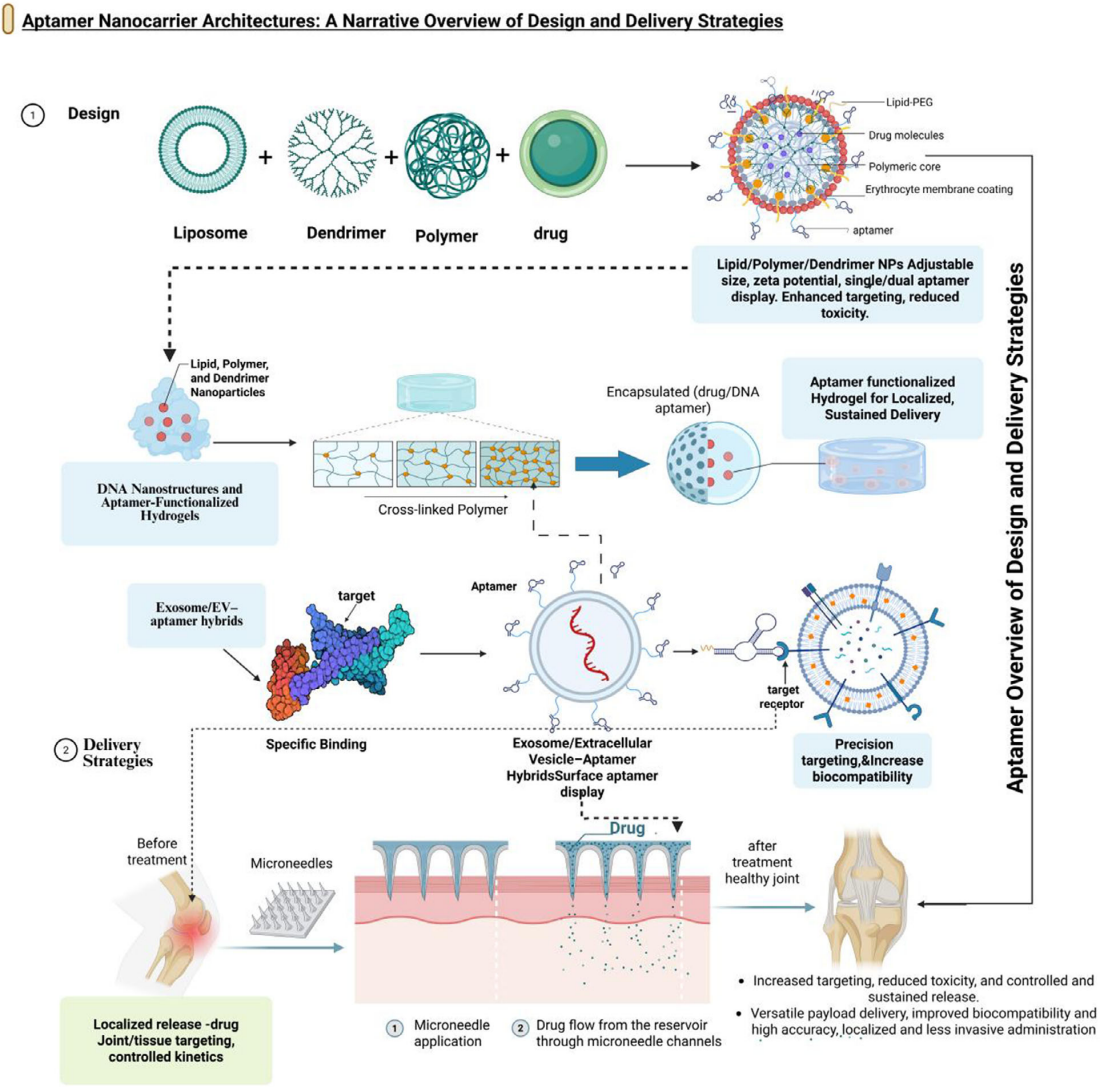
Aptamer nanocarrier architectures and delivery strategies for musculoskeletal targets. Overview of major nanocarrier architectures used for aptamer‐mediated delivery to bone and joint tissues. The upper tier depicts lipid, polymer, and dendrimer nanoparticles with tunable size, charge, and surface chemistry; DNA nanostructures and aptamer‐functionalized hydrogels that provide architected matrices for local release; and exosome/extracellular vesicle–aptamer hybrids offering biologically derived carriers with intrinsic tropism. The lower tier shows an exemplary microneedle or transdermal platform enabling localized release and controlled kinetics, ultimately directing the drug to the joint space. Together, these architectures constitute a modular toolbox for precision aptamer delivery in musculoskeletal indications. Illustrated architectures are supported by studies on lipid‐based aptamer systems for bone delivery, polymeric dual‐ligand nanoparticles [74], tetrahedral DNA nanostructures [50], aptamer‐functionalized hydrogels and scaffolds [52], exosome‐based delivery vehicles, and aptamer‐coated implant surfaces. This figure was created using BioRender.com.

Musculoskeletal aptamer systems are highly reliant on the carrier architecture, aptamer display, and ligand. carrier type influences the pharmacokinetics, tissue access, mechanical integration, and manufacturability of the engineered construct in bone, cartilage, synovium, marrow, and tumor compartments, whereas the conjugation chemistry and orientation determine whether a high‐affinity aptamer will be maintained in a functional form in the final construct. Carrier families, which are mostly used or proposed for bone and joint diseases, lipid‐based nanoparticles, polymeric particles and dendrimers, DNA nanostructures, hydrogels and scaffolds, biological and hybrid vehicles, and microneedle depots are typically summarized here (Figure 8) along with the influence of their design on the key aptamer variables of affinity, ligand density/valency, and spacer chemistry.

### 4.1 Lipid‐Based Systems for Bone and Joint Delivery

Liposomes and solid lipid nanoparticles (SLNs) are among the most sophisticated platforms for aptamer use. These are vital in musculoskeletal applications, primarily in bone tumors and skeletal metastases. They have flexible bilayered structures that allow both hydrophilic and hydrophobic payloads. The outer surface provides a chemically versatile surface for aptamers using PEGylated linkers, maleimide‐thiol conjugation, or click chemistry [64]. Aptamer‐coated liposomes and polymer‐lipid hybrids have been used to deliver cytotoxic agents and imaging probes to bone‐metastatic bone and breast cancer models, with bone lesions showing higher concentrations of aptamer‐liposomes and polymer‐lipid hybrids than aptamer‐ligand versions and higher bone: RES uptake ratios, resulting in better tumor control and reduced systemic toxicity [53, 99].

In the case of musculoskeletal cues, lipid‐based carriers are usually formulated in a size range of 80 150 nm, with a weakly negative zeta potential to optimize tumor or marrow penetration and escape excessive RES uptake [99]. Multivalent binding to receptors on the surface of liposomes can be directed to aptamers that target cancer stem cell markers (CD133 and EpCAM) or overexpressed receptors (nucleolin and integrins) with controlled densities, avoiding aggregation or complement activation [29]. Ideally, the same design logic would be applicable to purely orthopedic targets: osteoblasts, osteoclasts, or sclerostin aptamers on liposomes or SLNs can be used to deliver therapy to systemic areas of osteoporosis, and cartilage‐ or synovium‐targeting ligands may be used to support intraarticular liposomal depots that do not wash out rapidly and enhance retention in joint tissue [47, 94].

### 4.2 Polymeric Nanoparticles and Dendrimers, including Dual‐Ligand Constructs

Polymeric nanoparticles, including PLGA, PEG‐PLGA, and other amphiphilic block copolymers and dendrimers, have more stable structures and controllable degradation characteristics, which is why they are promising for sustained delivery in bone and joint diseases. Well‐known chemistries, such as carbodiimide, click chemistry, and thiol‐maleimide, can be used to densely functionalize the surfaces of these nanoparticles, and their cores can be loaded with chemotherapeutics, nucleic acids, or biologics [74, 104]. Aptamer‐functionalized polymeric nanoparticles with tumor receptor or CSC marker‐targeting properties have been shown to have better intratumor distribution, increased penetration, and better tumor regression in cancer models than nontargeted particles of the same construct [99].

The highly branched, monodisperse architecture of dendrimers is primarily used to control ligand valency. Bone‐targeting dendrimer systems with mineral‐affine peptides and aptamers must be optimized to achieve maximum ligand density. Excessive functionalization may lead to steric crowding, surface charge changes, and increased uptake by the liver, whereas moderate functionalization ensures maximum accumulation in the bone or marrow and reduces off‐target sequestration [74, 105]. In musculoskeletal applications, it is particularly important in narrow trabecular spaces or dense marrow, where over‐decorated particles cannot access owing to physical blockage. Hierarchical targeting dual‐ligand constructs that combine a bone‐homing aptamer (selected using SELEX) with a tumor‐ or synovium‐specific aptamer would be a promising solution to hierarchical targeting, in which initial targeting is in skeletal regions, and a tumor or inflamed population is selected subsequently [53, 99].

### 4.3 DNA Nanostructures and DNA‐Polymer Hybrid Systems

DNA nanostructures provide unrivaled precision in the aptamer space. DNA origami and other types of DNA nanostructures, such as tetrahedra called TDNs, can place multiple aptamer motifs at a distance and orientation with precision, allowing the systematic study of multivalency and cooperative binding [28, 106]. CH6‐functionalized TDNs in GelMA hydrogels have been utilized to precisely target osteoblast‐lineage cells and osteoinductive cues at the site of osteoporotic mandibular defects, resulting in better bone volume and mechanical strength than controls with no aptamers or scaffolds [47, 49]. TDNs can be used as structural scaffolds, multivalent aptamer scaffolds, and to deliver nucleic acid payloads simultaneously because TDNs are made of biocompatible DNA.

This design space is further expanded by hybrid DNA–polymer systems. For example, it can be conjugated or physically encapsulated within polymeric nanoparticles or hydrogels by exploiting the programmability of DNA and the mechanical strength and degradation characteristics of synthetic polymers [106, 107]. DNA frameworks that present chondrocyte or cartilage‐matrix aptamers might be tuned to the size and stiffness of pores to optimize their penetration of dense proteoglycan networks and retain aptamer accessibility [96]. From an engineering perspective, DNA nanostructures are best for the predictive modeling of aptamer spacing, valency, and conformational flexibility at biomaterial interfaces (Figure 8).

### 4.4 Hydrogels, Local Depots, and Scaffolds

Hydrogels and three‐dimensional scaffolds are the main forms of local depots used in musculoskeletal regeneration; however, they remain inert, and aptamer decoration is currently converting them into active‐targeting scaffolds. GelMA hydrogels with CH6‐TDN constructs reveal the mechanism by which a load‐bearing, cell‐friendly scaffold for bone defects improves through cell specificity during recruitment and signaling [49]. Surface‐conjugated Apt19S has been engineered to recruit native synovial and marrow‐based MSCs in anisotropic meniscal scaffolds, resulting in enhanced fibrocartilage deposition and meniscal structure in vivo [51]. More extensive studies with bispecific aptamers targeting synovial progenitors have been performed in large animals, where a single aptamer targets synovial progenitors and the other targets the scaffold, effectively forming a molecular bridge between the native niche and the defect [108].

Hydrogel chemistry and mechanics: Aptamers are highly dependent on these factors. The degradation rate, crosslinking density, and pore size are determinants of how aptamer‐decorated carriers and recruited cells migrate through the hydrogel via convection and diffusion. Nonspecific binding and aptamer stability are influenced by the local charge and hydrophobicity [40, 49]. Aptamers can be covalently conjugated to the hydrogel backbone via pendant groups, physically confined within the hydrogel, displayed on embedded nanoparticles, or identified as DNA nanostructures. All of them balance the criteria of stability, release, and accessibility to varying extents [28, 107]. In intraarticular depots in OA or RA, these matrices should endure repeated mechanical loading/shear at the cartilage‐synovium interface. Hence, the connection between aptamer chemistry and bulk mechanics is a key design feature (Figure 8).

### 4.5 Biological and Hybrid Vehicles: Exosomes, Cell‐Mediated Delivery, and Microneedles

Exosomes and extracellular vesicles (EVs) are biological agents with natural advantages, including biocompatibility and immunogenicity and the ability to cross certain biological barriers, thereby providing them with the potential to target musculoskeletal tissues. Nonskeletal models have displayed aptamers on EV surfaces via lipid insertion of aptamer‐PEG‐lipid conjugates, genetic fusion of aptamer‐binding domains, or covalent chemistry to direct vesicles to specific brain cells and tumors, which are better biodistributed than unmodified EVs [107, 109]. Various possibilities for potential translation of this technique to bone and joint diseases exist: mesenchymal stromal cell‐derived exosomes, adorned with CH6 or cartilage‐ matrix aptamers, could be concentrated in those regions of the body affected by osteoporosis or in degenerative cartilage; exosomes with synovial targets, such as IL‐6 or CTGF aptamers, can deliver anti‐inflammatory factors to the pannus [47].

There is a related notion of cell‐mediated delivery, in which aptamers cause homing or cargo release in living cells, not inert particles. Thus, aptamers that switch on the surface of developed T cells or macrophages could be developed to identify bone tumor epitopes and activate the local release of cytokines or cytotoxins. Nonetheless, these systems still theoretically exist in the musculoskeletal context [99]. More urgently, an aptamer‐conjugated microarray of microneedles and implant surfaces is a viable method for local, low‐invasive therapy. Drugs or nanoparticles can be delivered directly to the synovium in RA and OA using microneedle patches on the affected joints, providing greater precision and avoiding exposure of the system to a broad array of high‐exposure tissues. Incorporating synovial‐ or cytokine‐targeting aptamers into these systems can further localize therapy to the affected joint pannus and avoid cartilage and bone [21, 87]. In the case of prosthetic joint infections, aptamer‐functionalized surfaces on metal or polymeric components may allow high‐affinity capture of bacteria and the local application of systemic antibiotic therapies [87].

### 4.6 Chemistry and Orientation Control

In any carrier class, the chemistry used to attach aptamers to nanocarriers or matrices is an important factor in determining their functionality. Random multipoint conjugation may disrupt the aptamer secondary structure, occlude binding sites, and yield heterogeneous products that are difficult to characterize and maintain. Site‐directed techniques allow the control of the orientation, density, and length of linkers, which can be aligned with the design variables noted in Section 2 [107, 110].

Conventional techniques involve thiol‐maleimide conjugation of 5′‐or 3^′^‐thiolated aptamers to maleimide‐containing lipids, polymers, or scaffold backbones; azide–alkyne cycloaddition (also known as click chemistry); copper‐catalyzed or strain‐promoted azide‐functionalized carriers and alkyne‐functionalized aptamers; or carbodiimide‐mediated (EDC/NHS) connection of amine‐functionalized molecules. Streptavidin‐biotin interactions are highly affine; however, they introduce protein elements that may increase the complications in immunogenicity and CMC. In musculoskeletal systems, where chronic administration or permanent implants are proposed, there is a solid reason to favor full synthetic, covalent bonding with clear stoichiometry and a low immune foot sample [46].

Conjugation must be considered with respect to spacer design. Although correctly oriented aptamers are survival‐viable, overly short or stiff linkers may pose a constraint on conformational flexibility, particularly on surface crowding or in a dense matrix, such as cartilage, despite a favorable Kd in solution [110]. 112 In contrast, very long or loose polymers can cause an enlargement in hydrodynamic size, a change in zeta potential, or expose the aptamer to proteins. In both systematic studies of electrochemical aptasensors and targeted nanocarriers, middle‐range spacer lengths are usually found to be the most beneficial in terms of signal and binding performance, which ought to be more explicitly designed and reported in the context of bone and joint applications [74, 110].

In conclusion, with these two architectural and conjugation inventions, a viable plan for designing based on musculoskeletal aptamer therapeutics (Figures 5 and 6) will be plotted. Systemic delivery in bone tumors and osteoporosis is achieved using lipid and polymeric nanoparticles. Spatial control of defect repair and cartilage or meniscus regeneration using DNA nanostructures, hydrogels and scaffolds, exosomes, microneedles, and coatings offers prospects for nanoparticle use. Success on all these platforms requires strict control and open reporting on aptamer orientation, density, spacer chemistry, and carrier physicochemical properties so that the field can extend beyond presumed prototypes to reproducible, translatable platforms.

## 5 Routes of Delivery, Biodistribution, and Retention

**FIGURE 9:**
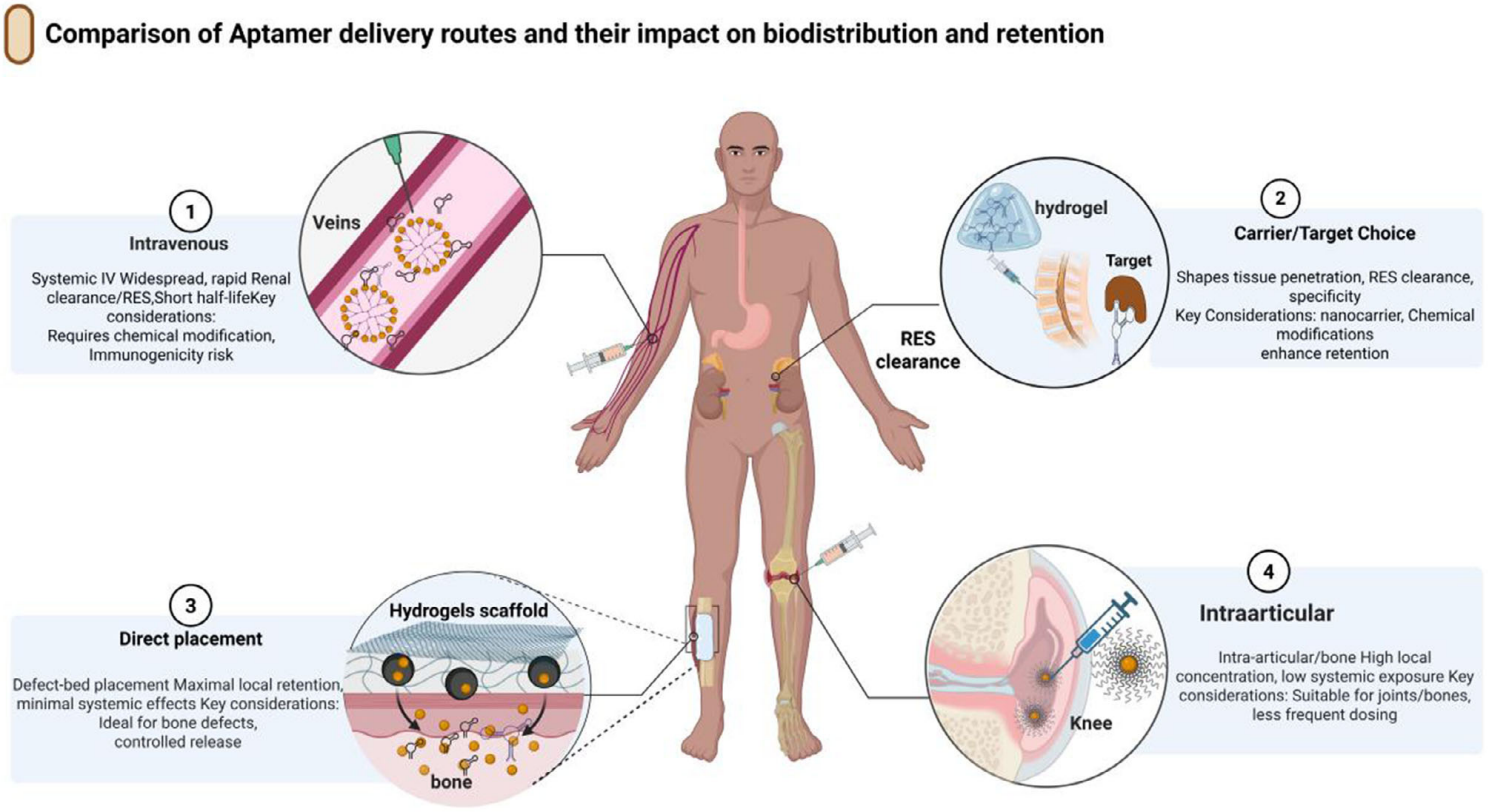
Routes of administration for aptamer‐based musculoskeletal therapeutics. Comparative overview of delivery routes for aptamer‐guided therapy. The central anatomy highlights representative injection sites, while the surrounding panels summarize: (1) intravenous systemic administration, which provides widespread exposure but rapid renal and reticuloendothelial clearance and short half‐life, necessitating chemical stabilization and careful management of immunogenicity; (2) carrier‐ and target‐optimized delivery, where selection of nanocarrier and target site (for example, hydrogel–bone interface) shapes tissue penetration, specificity, and retention; (3) direct placement, involving defect‐bed or peri‐osseous implantation of aptamer‐functionalized hydrogels or scaffolds to maximize local retention and minimize systemic exposure; and (4) intraarticular injection, achieving high local concentrations within joints and low systemic levels, particularly suited to large joints such as the knee and allowing less frequent dosing. Route selection is informed by intravenous bone‐targeted delivery [53], local intraarticular injection for joint retention, defect‐bed implantation of aptamer‐scaffold constructs [52], and trans‐synovial microneedle delivery for periarticular drug access. This figure was created using BioRender.com.

The delivery route determines the migration of aptamer‐functionalized nanocarriers from the circulation or local stores to bone, cartilage, synovium, and marrow, as well as their retention in the latter. For musculoskeletal indications, systemic intravenous (IV) administration is the most common route for treating osteoporosis and bone metastases. Local delivery methods include intraarticular injection of joints, defect‐bed implantation of foci, and new microneedle‐based administration methods for recombinant drugs into joints and periarticular spaces (Figure 9). The biodistribution and pharmacokinetics of these routes should be measured based on musculoskeletal‐specific measures, including to bone‐to‐reticuloendothelial system (bone: RES) ratios and joint retention to comparative systemic exposure [28].

### 5.1 Osteoporosis and Bone Metastases

Systemic intravenous (IV) administration is the most viable approach for managing diffuse bone disorders, including osteoporosis and metastatic diseases of both the axial and appendicular skeletons. Nanocarriers targeted to bones and aptamers targeted to them, selected in vivo by SELEX, have been shown to specifically accumulate in skeletal lesions rather than in the liver and spleen, thereby enabling bone: RES ratio enhancement compared with untargeted nanoparticles [53]. In such systems, aptamers act as “address tags” for conventional bone‐seeking chemistries or for size‐ and charge‐tuned carriers, thereby restoring a larger fraction of the injected dose to the trabecular and cortical regions. On a translational scale, the systemic delivery of aptamers is beneficial for multifocal diseases when repeated dosing is required and the safety of the system is undergoing routine evaluation, including liver, kidney, and marrow conditions [29].

In musculoskeletal applications, aptamer–nanocarrier systems must penetrate multiple barriers, including plasma proteins, endothelial walls, marrow sinusoids, and, in certain instances, pathologic neovessels in tumors, to achieve systemic delivery. The design space in which pharmacokinetic optimization occurs is a well‐defined but strict design space: a hydrodynamic size of between 50–150 nm to avoid rapid renal clearance and slow filtration by the spleen, surface PEG or other stealth‐targeted analogs to extend circulation time, and aptamer valency is set to optimize bone or tumor targeting, yet must not result in rapid opsonization and aggregation [28, 111], aptamers with high affinity to a target in vivo (such as bone‐homing ligands in metastatic models) are more likely to retain targeting activity under shear forces and at protein corona formation when exposed to systemic delivery [29, 53, 112].

### 5.2 Local Intraarticular Injection, Defect‐Bed Implantation, and Peritumoral Delivery

In contrast, local delivery techniques are more feasible because of the numerous musculoskeletal indications that are focused on and mechanically limited. The use of aptamer‐coated nanoparticles, DNA nanostructures, or hydrogels injected directly into the joint space has been relevant for intraarticular injection in arthritis (such as osteoarthritis), RA, and meniscal conditions. They do not require clearance of the entire body before entering the system, reduce the time spent in systemic circulation, and allow the carrier to target cartilage penetration and synovial retention instead of long circulation times [28]. Particles smaller than 100 nm, comprising flexible PEG spacers and near‐neutral or slightly negative surface charge, have been shown to reside better in cartilage and the synovium, particularly when coupled with chondrocyte‐ or synovial‐homing aptamers [96, 107].

The first is defect‐bed implantation, which is used for segmental bone defects and meniscal repair. In this method, aptamer‐functionalized scaffolds, hydrogels, or hybrid DNA‐polymer depots are inserted into the defect during surgery and constitute a permanent reservoir that delivers growth factors, small molecules, or nucleic acids on site. For example, to regenerate osteoporotic mandibles, CH6‐functionalized tetrahedral DNA nanostructures immobilized within GelMA hydrogels were inserted into defects to induce osteoblast migration and enable local osteogenesis, with systemic exposure primarily restricted to degradation products [49]. Aptamer‐functionalized carriers have also been investigated in models of peri‐tumoral injection or implantation in osteosarcoma and bone metastasis, enabling high local drug concentrations and reduced systemic toxicity, particularly in combination with cytotoxic or ICD‐targeting cargoes [29, 99].

### 5.3 Trans‐Synovial (periarticular) Delivery Using Microneedles

The microneedle platform provides a solution between local injections and systemic delivery. Drugs can be delivered through the periarticular space as intraarticular injections, which often cause discomfort and carry a risk of infection. However, arrays can be placed over or near the diseased joint to deliver drugs into the space between the joints and the surrounding subcutaneous tissue. Aptamers can be added to microneedle surfaces or nanoparticles released by dissolving microneedles, and new aptamers targeting synovial fibroblasts, cytokines, or cartilage surfaces can, in principle, target payloads to inflammatory pannus or degeneration in cartilage surfaces rather than periarticular fat or muscle [107, 113].

In our opinion, microneedle‐based delivery of aptamers simplifies the paradoxes of carrier stability in the blood but introduces new challenges in mechanical strength, depth of insertion, and release dynamics. Biodegradable polymer needles must remain mechanically strong throughout their use but dissolve or erode in a predictable manner, and aptamers must retain their structure during fabrication and storage. The first signs of nonmusculoskeletal effects using analogs of microneedle‐delivered nanoparticles suggest that favorable local‐to‐systemic ratios of nanoparticles and uniform pharmacokinetics can be achieved by this technique [107, 109] earlier trials. The different routes of intraarticular, defect‐bed, microneedle, and systemic administration are schematically summarized in Figure 9 to demonstrate that these routes differ in their effects on biodistribution.

### 5.4 Biodistribution Metrics and Pharmacokinetics: Bone, RES Index, and Joint Retention

Regardless of the route, it is rational to optimize aptamer‐functionalized systems with musculoskeletal‐specific biodistribution metrics. In the case of systemic bone‐targeted therapies, the bone: RES index, typically defined as the ratio of aptamers to nanocarriers in bone (or bone lesions) n to that in the liver and spleen, is a useful measure of targeting efficacy [95]. In vivo SELEX‐derived bone‐homing aptamers have demonstrated higher bone: RES ratios than untargeted nanoparticles, and the rates correlate with better lesion containment and reduced off‐target toxicities in metastatic disease models [53]. In engineering terminology, the bone‐to‐RES ratio is a cumulative metric that quantifies the impact of size, surface chemistry, aptamer affinities/valency, and circulation half‐life.

Vital parameters of intraarticular and periarticular delivery, joint retention, and systemic leakage were assessed. Synovial and cartilage retention half‐lives can be measured using labeled aptamer‐nanocarriers, and systemic exposure to plasma and organ biodistribution can be measured using serial imaging or sampling of synovial fluid [28, 96]. The most suitable designs are those with the greatest joint area under the curve (AUC) and the least plasma AUC and hepatic/spleen uptake, because efficacy is local in osteoarthritis and RA. However, systemic adverse events, such as immunosuppression or hepatotoxicity, must be prevented. Other measures, such as the bacterial loading of the bone versus soft tissues or biodepot washout kinetics, are involved in the case of infections and osteomyelitis, mainly when antibiotic payloads are delivered to bone conversions or biofilm‐coated hardware through the use of bacterial aptamers [87].

Pharmacokinetic models generated by oncology systems and gene therapies now include compartments for the bone marrow, cortical bone, and synovial fluid, enabling simulation‐based analysis of the impact of variations in aptamer affinity, valency, or carrier size on the peaks in musculoskeletal tissues and their exposure kinetics. Orthopedic platforms guided by aptamers should be the next generation of models, in which standards and reports are straightforward whenever aptamers are used [29].

#### 5.4.1 Lessons From the Nonmusculoskel etal Aptamer Biodistribution

As most musculoskeletal aptamer–nanocarrier systems have not reached the proof‐of‐concept stage for many applications, their designs must be adapted with caution for more advanced applications, such as oncology, neurology, and infectious diseases. Some of the underlying principles have already been established through aptamer‐based nonbone tumor systemic therapy and aptamer‐based nanoparticles for brain delivery, both developed for such solid tumors. First, it does not mean that high‐affinity aptamers do not need to grapple with the issue of unwanted size, charge, or stealth properties; successful tumor‐targeted systems are optimized at the Nanometer scale, and aptamer density and orientation are designed with care [28, 29, 107]. Second, the surest way to increase the circulation time is to coat the stealth with a coating, such as poly (ethylene glycol) (PEG). However, the presence of anti‐PEG antibodies and hypersensitivity reactions prior to drug intake adversely impacts the safety and clearance of PEGylated drugs [111]. Third, based on exosome‐ and nanovesicle‐based carriers, biologically derived membranes can be exploited to positively influence biodistribution; however, issues of ligand presentation and batch‐to‐batch reproducibility become defining CMC concerns [109].

To translate to musculoskeletal, the above lessons awaited imply that the bone‐ and joint‐targeting aptamer platforms will have to: (i) further provide detailed physicochemical and surface‐ligand characterization and biodistribution, (ii) embed immunogenicity monitor of PEG or other stealth polymers in chronic diseases such as osteoporosis and RA, and (iii) address biological carriers (such as exosomes) when a manufacturing and quality‐control facility can assure reproducible ligand

## 6 Harmonizing Efficacy Readouts Across Indications

**FIGURE 10:**
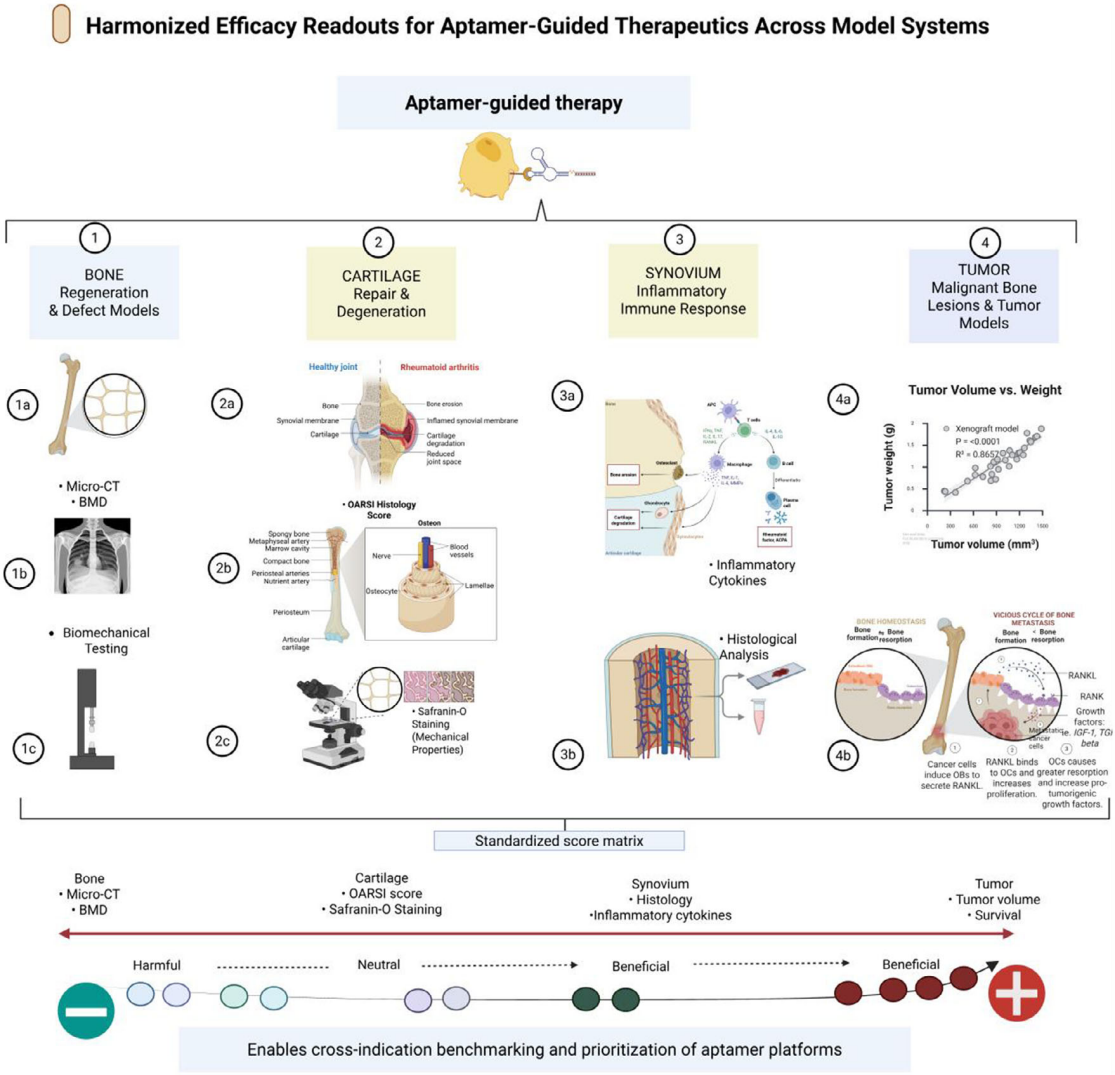
Harmonized efficacy readouts for aptamer‐guided therapeutics in bone, cartilage, synovium, and tumor models. Integrated view of outcome domains and cross‐domain effect‐direction analysis for aptamer‐based interventions. The upper panel summarizes the domain‐specific endpoints: micro‐CT‐derived bone regeneration metrics, BMD, trabecular architecture, and biomechanical testing in bone and defect models; OARSI histology scores, safranin‐O staining, and mechanical indentation tests in cartilage repair; histological assessment and inflammatory cytokine profiling in synovial inflammation; and tumor volume, survival, metastatic burden, and bone integrity in malignant bone lesions. The lower panel depicts effect‐direction plots that enable a visual comparison of treatment effects across bone, cartilage, synovium, and tumor outcomes, facilitating cross‐indication benchmarking and prioritization of aptamer platforms. Outcome domains are based on micro‐CT and BMD measurements for bone regeneration [49], OARSI histological scoring and proteoglycan quantification for cartilage repair, synovial inflammation indices for arthritis [85], and tumor volume and survival metrics for skeletal malignancies. This figure was created using BioRender.com.

Preclinical aptamer‐nanocarrier analyses of bone and joint diseases have shown a wide range of structural, functional, and biological outcomes. To compare different indications and platforms, efficacy must be evaluated using standard domain‐specific views of the bone, cartilage/meniscus, synovium/inflammation, tumor, and infection. The current literature on benefits in domains and projects and how these readouts can be subset into a comprehensible interpretive taxonomy is described here (Figure 10).

### 6.1 Bone Outcomes

The most widely used multimodal evaluation of bone‐targeted aptamer systems integrates microcomputed tomography (microCT), BMD assessment, and biomechanical testing. MicroCT provides high‐resolution structural readouts and should be reported using standardized parameters and acquisition conventions, including trabecular bone volume fraction (BV/TV), trabecular thickness (Tb.Th), trabecular number (Tb.N), trabecular separation (Tb.Sp), connectivity density, and cortical thickness, to enable cross‐study comparability in rodent models [114]. BMD is most commonly quantified by dual‐energy X‐ray absorptiometry or, when volumetric density is required, by quantitative CT, with careful attention to positioning and region‐of‐interest definition to maintain precision in small‐animal studies [115]. Functional integrity is then validated by mechanical testing, typically three‐point bending, compression, or torsion, reporting stiffness, ultimate load, and energy‐to‐failure at the defect or osteoporotic site under standardized small‐animal testing frameworks [116]. Within this framework, aptamer‐functionalized constructs, such as CH6‐decorated systems embedded in regenerative depots, can be evaluated for convergent structural and functional benefits, where improvements in microarchitecture and mineralization are accompanied by higher mechanical competence relative to nontargeted carriers or scaffold‐only controls [49].

### 6.2 Cartilage and Meniscus Outcomes

As primary structural measures, the Osteoarthritis Research Society International (OARSI) histopathology scores and relevant systems for evaluating focal defects are used for cartilage and the meniscus. They are semi‐quantitative scales used to determine the levels of cartilage surface integrity, lesion depth and extent, loss of matrices, and chondrocyte changes, and have been validated in over one species of tissue (human) and in small‐animal models [117, 118]. Proteoglycans, a biochemical marker of cartilage health, are usually visualized with safranin O stain or toluidine blue and measured by optical density or scoring. The content of proteoglycans, which is commonly used as evidence to demonstrate that aptamer‐treated joints are retained/repaired. Other measures of functional recovery under load, including mechanical indentation stiffness, represent important constructs for Apt19S‐decorated meniscal scaffolds or synovial‐bridging bispecific aptamers. Integration measures, defect occupation, interface continuity, and subchondral bone remodeling can be assessed histologically or by uCT, which shows an anatomical perspective; in other words, between structural repair and superficial coverage [117, 118].

### 6.3 Synovial and Inflammatory Outcomes

Aptamer‐based interventions in preclinical RA and related inflammatory arthritis models are typically evaluated using complementary readouts that capture clinical disease activity, inflammatory burden, and tissue destruction. Disease activity is most often tracked longitudinally using a composite clinical arthritis score that integrates paw or joint swelling, erythema, and loss of function or deformity and is frequently paired with objective caliper‐based measurements of paw or ankle thickness to quantify edema with higher resolution and reduced observer subjectivity [119, 120, 121]. Pharmacodynamic efficacy is then anchored to pathway biomarkers, including circulating and/or joint cytokines and chemokines, which are particularly relevant for mechanism‐directed aptamers such as IL‐6 antagonistic aptamers and CTGF‐targeting [122, 123, 124]. Histopathology provides the definitive structural endpoint, with semi‐quantitative scoring frameworks that encompass synovitis and lining‐layer hyperplasia, pannus formation and angiogenesis, and the extent of cartilage and bone erosion; standardized microscopic scoring recommendations have been developed specifically to improve reproducibility and reporting quality across inflammatory arthritis models [125]. Across studies, concordant improvements in clinical scores and caliper‐measured swelling, normalization of inflammatory mediators, and attenuation of pannus‐driven erosive pathology collectively indicate the therapeutic benefit of aptamer‐enabled strategies in inflammatory arthritis, although rigorous head‐to‐head comparisons with established disease‐modifying regimens and harmonized outcome reporting remain essential [85, 122, 126].

### 6.4 Tumor and Infection Outcomes

Aptamer‐based interventions in preclinical RA and related inflammatory arthritis models are typically evaluated using complementary readouts that capture clinical disease activity, inflammatory burden, and tissue destruction. Disease activity is most often tracked longitudinally using a composite clinical arthritis score that integrates paw or joint swelling, erythema, and loss of function or deformity, and is frequently paired with objective caliper‐based measurements of paw or ankle thickness to quantify edema with higher resolution and reduced observer subjectivity [119, 120, 121]. Pharmacodynamic efficacy is then anchored to pathway biomarkers, including circulating and/or joint cytokines and chemokines, which is particularly relevant for mechanism‐directed aptamers such as IL‐6 antagonistic aptamers and CTGF‐targeting aptamers [85, 122, 123]. Histopathology provides the definitive structural endpoint, with semi‐quantitative scoring frameworks that encompass synovitis and lining‐layer hyperplasia, pannus formation and angiogenesis, and the extent of cartilage and bone erosion; standardized microscopic scoring recommendations have been developed specifically to improve reproducibility and reporting quality across inflammatory arthritis models [125]. Across studies, concordant improvements in clinical scores and caliper‐measured swelling, normalization of inflammatory mediators, and attenuation of pannus‐driven erosive pathology collectively indicate the therapeutic benefit of aptamer‐enabled strategies in inflammatory arthritis, although rigorous head‐to‐head comparisons with established disease‐modifying regimens and harmonized outcome reporting remain essential [85, 122, 126].

### 6.5 Integrating Diverse Readouts into a Clear Framework

Across bone, cartilage/meniscus, synovium/inflammation, tumor, and infection applications, outcome reporting remains too heterogeneous to support meaningful quantitative synthesis (Figure 10). Studies vary substantially in species, disease induction, anatomical site, carrier class, dosing regimen, and follow‐up windows, and even when the same endpoint is named (such as BV/TV, OARSI score, arthritis grade, tumor volume, bacterial burden), it is often measured on different scales or reported semi‐quantitatively rather than as fully extractable numerical data [114, 117, 118]. In addition, many reports remain proof‐of‐concept and emphasize representative images or percentage changes without complete tabulated datasets, while core internal‐validity safeguards (randomization, blinding, prespecified handling of attrition) are inconsistently documented, limiting interpretability and cross‐study comparability [87, 123]. Under these conditions, statistical pooling is not only methodologically fragile but also risks producing biologically misleading conclusions.

A more defensible synthesis strategy at this stage is structured evidence mapping using harmonized outcome domains, as schematized in Figure 10, which can reveal design‐relevant signals without over‐interpreting sparse or nonuniform data [127, 128]. Concretely, aligning future studies to established measurement frameworks, such as rodent bone micro‐CT reporting standards and OARSI histopathology systems for cartilage and synovium, would immediately increase reproducibility, enable consistent extraction of core endpoints, and create the conditions required for rigorous meta‐analyses that genuinely inform engineering decisions and translational prioritization [114, 117, 118].

## 7 Engineering Design Rules and Performance Targeting

The design variables focused on for aptamer‐functionalized nanocarriers for the treatment of bone and joint diseases include particle size and distribution, surface charge, ligand density and valency, spacer architecture, and cellular, matrix, or pathway targets. Considering the systems in Table 2 and the aptamer panel in Table 1, apparent vacant spaces that guide further design become apparent, rather than new groups rediscovering previously tested empirical principles. Here, we generalize these patterns of structure–function, propose quantitative measures for targeting performance, and describe decision schemes based on specific indicators. An overview of the important aptamer design variables is provided in Figures 1, and 9 combines them into an engineering decision‐making schematic.

### 7.1 Extracting Design Windows From Existing Musculoskeletal Systems

#### 7.1.1 Design Windows Extraction of Design Windows of Existing Musculoskeletal Systems

In musculoskeletal delivery, most aptamer‐functionalized nanocarriers that report efficient access to bone marrow, trabecular compartments, or skeletal lesions cluster within a relatively narrow physicochemical window. Across bone‐targeted liposomes, polymeric nanoparticles, and related systems, reported hydrodynamic diameters frequently fall in the 80–150 nm range, with low dispersity (often PDI <=0.2–0.3) and mildly negative to near‐neutral zeta potentials, reflecting a practical compromise between vascular transport, extravasation, marrow penetration, and avoidance of rapid opsonization and clearance [53, 129, 130]. This design logic is consistent with recent bone‐targeting implementations that use aptamers as cell‐selective ligands on nanocarriers to bias biodistribution and cellular uptake in osteoporosis‐relevant settings [131] and with broader aptamer‐nanoparticle design frameworks used in musculoskeletal and oncology‐adjacent contexts [95, 99]. By contrast, cartilage‐accessing constructs are generally engineered smaller (commonly under ∼ 80–100 nm) and rely on compliant PEG‐like spacers to navigate the dense glycosaminoglycan network while limiting nonproductive electrostatic trapping; these constraints are repeatedly emphasized in cartilage‐targeting delivery analyses and are aligned with aptamer‐enabled chondrocyte targeting in osteoarthritis models [47, 96, 132]. Importantly, while cationic surfaces can improve nucleic‐acid complexation, they also increase protein adsorption, reticuloendothelial uptake, and inflammatory/toxicity liabilities in vivo, reinforcing the need for charge moderation in joint‐ and marrow‐facing formulations [48, 95].

Ligand density and valency show a similarly constrained ‘design window.’ Across targeted nanomedicine, cellular binding and uptake typically improve as ligand density increases up to an intermediate regime, after which steric crowding, conformational restriction, and particle–particle interactions begin to dominate, and performance can plateau or decline, producing a bell‐shaped response curve [64, 75, 133]. Although musculoskeletal aptamer reports do not consistently quantify ‘aptamers per particle’ (or per unit surface area), successful systems implicitly sit within this intermediate regime, as suggested by bone‐homing selections and bone‐targeted nanocarriers [53, 131]. Moreover, scaffold‐ or DNA‐nanostructure presentations that exploit multivalency without forcing nanoscale crowding [49, 51]. Practically, DNA nanostructures and anisotropic scaffolds can distribute aptamer motifs over larger length scales, enabling avidity gains while reducing aggregation risk relative to densely grafted nanoparticle coronas [49, 51].

The spacer‐and‐linker architecture is the third recurring determinant of performance. Surface immobilization can suppress aptamer folding and accessibility when linkers are too short or when grafting density is high; conversely, overly long polymeric spacers can increase hydrodynamic size and alter interfacial behavior, so intermediate linker lengths that preserve conformational freedom while maintaining controlled presentation are repeatedly favored in systematic studies of immobilized aptamers and surface binding [77, 133, 134]. In musculoskeletal terms, cartilage‐facing platforms typically benefit from longer, more compliant spacers to reach cellular epitopes through matrix barriers, whereas bone marrow or tumor‐facing systems can tolerate shorter linkers when epitopes are more exposed and the towardsity exposed and shifts towards maintainingcompact size and favorable pharmacokinetics [53, 130, 132]. Collectively, the systems summarized in Table 2 support three actionable design rules for musculoskeletal targeting: (i) systemic bone/tumor delivery most often converges on ∼80–150 nm carriers with mildly negative or near‐neutral surfaces; (ii) cartilage/meniscus access generally demands smaller constructs (often under ∼80–100 nm) with flexible spacers to maintain accessibility within dense matrix; and (iii) local depots and larger substrates can exploit avidity via spatially distributed multivalency while minimizing nanoparticle aggregation penalties (Figures 1 and 2) [47, 49, 51, 132].

### 7.2 Quantitative Indices for Bone Targeting and Joint Retention

Qualitative claims, such as ‘enhanced bone uptake’ or ‘extended joint residence, ’ are insufficient for engineering optimization or quantitative comparison between platforms. A step toward standardization is to define a bone‐to‐RES index, calculated as the ratio of the percentage of injected dose (%ID) in bone or bone lesion to the corresponding %ID in the liver and spleen at the same time point. Bone‐homing aptamer–nanoparticle systems developed in oncology contexts have demonstrated marked improvements in this index, reflecting preferential skeletal accumulation and reduced hepatic/splenic deposition, compared with untargeted nanocarriers [53, 99]. Applying this quantitative benchmark to nonmalignant bone conditions, such as osteoporosis or osteomyelitis, could establish explicit translational thresholds for targeting efficiency, moving beyond qualitative imaging or histologic descriptions (Figures 1, 2).

For intraarticular applications, a complementary joint retention index can be formulated by comparing systemic and local exposure, specifically, the ratio of plasma area under the curve (AUC) to the AUC measured in synovial fluid or cartilage following intraarticular administration. Controlled‐release liposomal and polymeric depots have already shown significant improvements in joint AUC with concomitant reductions in systemic drug exposure in osteoarthritis and RA nanomedicine models [135, 136]. Decorating such carriers with cartilage‐ or synovium‐specific aptamers is expected to further minimize washout, enhance local retention, and improve therapeutic ratios [96, 123]. Standardizing the reporting of bone‐to‐RES and joint‐retention indices across aptamer studies would convert targeting performance from a qualitative narrative into quantitative engineering metrics that can be compared, optimized, and used to define go/no‐go criteria (Figures 1 and 2).

### 7.3 Comparison of Ligand Classes and Valency Strategies

Table 1 categorizes musculoskeletal aptamers into cell‐, matrix‐, pathway‐, and pathogen‐targeting classes, each with distinct designs and functional implications. Cell‐targeted aptamers, such as CH6 (osteoblast‐lineage specific) and Apt19S (mesenchymal stromal cell‐specific), have demonstrated efficacy in spatially controlled regeneration systems, for example, CH6‐functionalized tetrahedral DNA nanostructures embedded in GelMA hydrogels enhanced osteoblast recruitment and bone formation in osteoporotic mandibular defects [49], and Apt19S‐functionalized anisotropic meniscal scaffolds improved fibrocartilage regeneration via endogenous stromal cell recruitment [51]. Matrix‐targeted aptamers, including hydroxyapatite‐binding ligands, facilitate depot anchoring and mineral‐surface adhesion, enhancing cell–matrix interaction and drug retention at bone interfaces [137, 138]. Pathway‐targeted aptamers (such as anti‐sclerostin, anti‐IL‐6, or anti‐CTGF) act both as targeting motifs and as functional regulators of osteoanabolic and inflammatory signaling, providing dual therapeutic value [85, 123, 139]. Finally, pathogen‐targeted aptamers are being developed to selectively bind bacterial surface antigens, such as those on Staphylococcus aureus, for applications in osteomyelitis and prosthetic joint infection, supporting targeted antimicrobial delivery or diagnostic capture [87].

While most musculoskeletal systems employ single aptamers, recent work in cancer nanomedicine and biosensing highlights the potential of dual‐aptamer and logic‐gated (AND‐gate) designs to enhance specificity and reduce off‐target activity [99, 107]. Dual‐target architectures that combine a bone‐homing aptamer with a tumor‐ or CSC‐specific aptamer have improved skeletal targeting in metastasis models, and similar bispecific constructs linking synovial progenitors and meniscal tissue have achieved functional repair in large‐animal studies [51, 135]. Ultimately, the ligand class and valency strategy must align with therapeutic intent: cell‐targeted, multivalent constructs for regeneration and immune modulation; matrix‐targeted ligands for depot anchoring and long‐term spatial control; and multiaptamer or logic‐gated systems for differential recognition between diseased and healthy tissue compartments (Figures 1, 9 and Table 1).

### 7.4 Indication‐Specific Decision Structures and Architectures

In terms of engineering, the currently available systems support a practical, indication‐specific decision framework (Figure 9). For osteoporosis and focal bone defects, the most defensible approach is to combine (i) a bone‐homing or osteoblast‐targeting aptamer (for example, bone‐homing ligands enriched by in vivo selection or the osteoblast‐binding CH6 aptamer), (ii) a nanoparticle or DNA nanostructure platform with moderate multivalent aptamer display and low absolute zeta potential (often within an 80–150 nm hydrodynamic window for systemic constructs), and (iii) either systemic dosing designed to maximize bone‐to‐reticuloendothelial system partitioning or local incorporation into a slowly degrading hydrogel or scaffold to resist inflammatory and mechanical stresses [44, 49, 53] (Figure 3). For cartilage and meniscus, effective constructs tend to prioritize (i) mesenchymal stromal cell recruiting or cartilage/matrix‐directed aptamers, (ii) either sub‐100 nm carriers or porous scaffolds using flexible linkers and neutral‐to‐slightly‐negative surface charge to preserve ligand accessibility within dense extracellular matrices, and (iii) intraarticular delivery with endpoints anchored to histologic, compositional, and mechanical restoration readouts [51] (Figure 4). For synovitis‐driven diseases, the most rational near‐term strategy is to optimize (i) synovial‐homing or pathway‐targeted ligands (for example, anti‐IL‐6 aptamers) and/or (ii) local depot formats such as hydrogels or microneedle‐enabled intraarticular delivery that concentrate exposure within the pannus while limiting cartilage and bone off‐target burden; however, most integrated ‘aptamer plus advanced depot’ RA systems remain developmental and require systematic validation in standardized models [107, 122].

In bone tumors and skeletal metastases, the most mature design logic is hierarchical targeting that couples (i) tumor‐associated or cancer stem cell‐directed aptamers with (ii) bone‐homing/lesion‐enrichment behavior and (iii) cytotoxic, immunogenic‐cell‐death, or theranostic payloads, implemented on clinically scalable lipid or polymer nanocarriers and benchmarked using lesion uptake and survival‐relevant outcomes [95, 107] (Figure 7). Finally, for infection and osteomyelitis, the most viable near‐term solution is predominantly local: pathogen‐targeted aptamers paired with antibiotic‐loaded nanoparticles, hydrogels, or implant coatings to enhance binding and biofilm engagement at infected bone/implant interfaces and sustain local exposure over time [86, 87]. Collectively, these strategies are deliberately conservative; they do not exhaust the design space but distill what has reproducibly worked across models into a translational rule set that can be progressively converted into quantitative design maps linking size, charge, ligand density, and target class to biodistribution indices, joint retention, efficacy endpoints, and safety profiles (Figure 9; Table 1).

## 8 Safety, Immunogenicity, and Biocompatibility

### 8.1 Intrinsic Safety Profile of Nucleic Acid Aptamers

**FIGURE 11:**
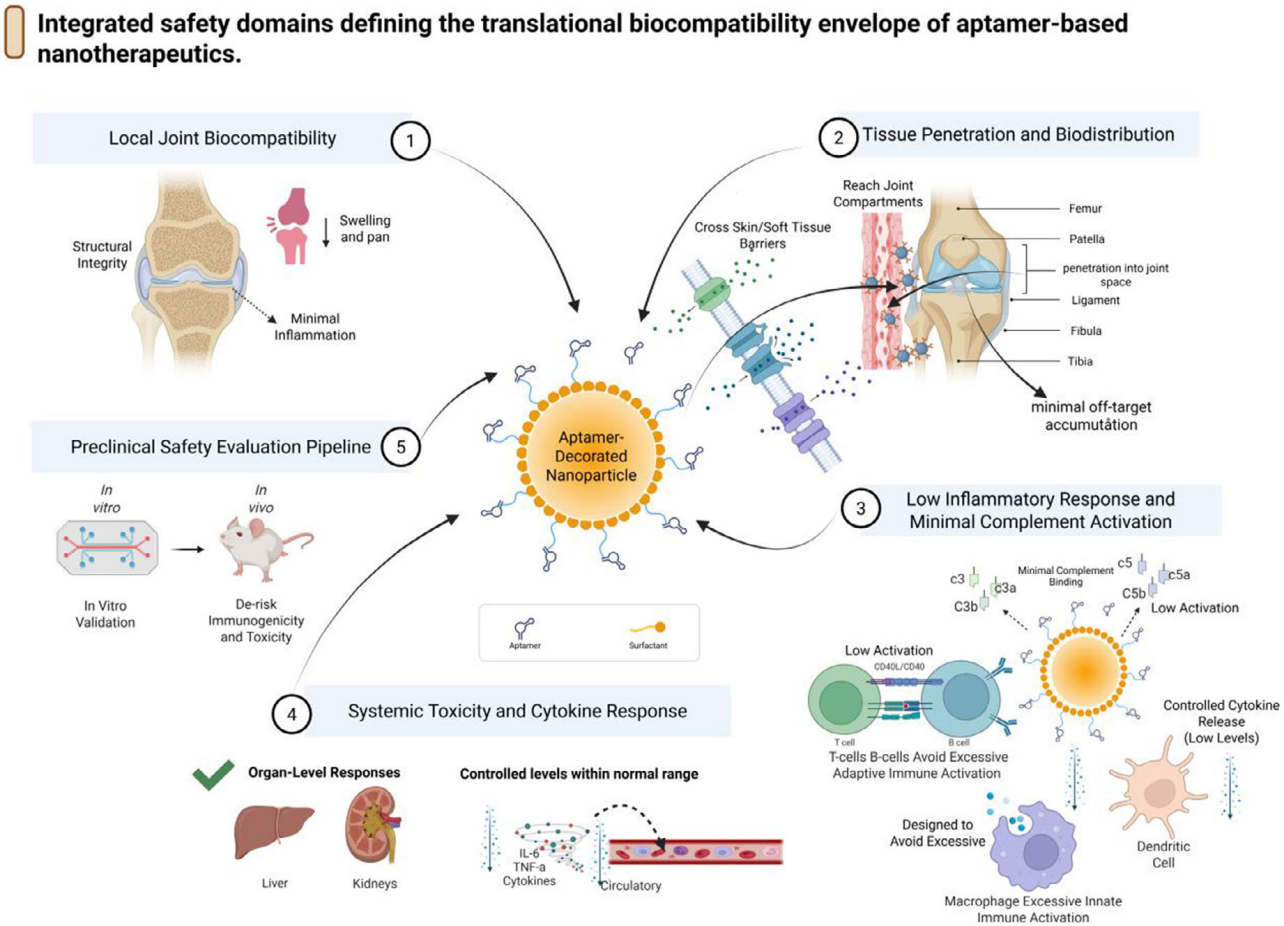
Safety, immunogenicity, and biocompatibility landscape of aptamer‐based therapeutics. Radial framework summarizing the key safety and biocompatibility domains for aptamer platforms. The central panel represents an aptamer‐decorated nanoparticle surrounded by domains encompassing joint tissue response (local tolerance within cartilage, synovium, and periarticular tissues, including swelling, pain, and structural integrity), tissue penetration and distribution (ability to cross skin or soft‐tissue barriers and reach joint compartments without problematic off‐target accumulation), low inflammatory response and minimal complement activation (designed to avoid excessive innate or adaptive immune activation), systemic effects (organ‐level responses in the liver, kidney, and other systems and systemic cytokine release), and in vitro/in vivo assays (tiered testing from cell culture to animal models to de‐risk immunogenicity and toxicity). Collectively, these domains define the safety envelope required for the translation of aptamer therapeutics. Safety considerations are drawn from aptamer‐specific immunogenicity data, including anti‐PEG antibody responses [28], complement activation by phosphorothioate backbones, and local tissue compatibility assessments in bone, cartilage, and marrow environments [91, 92]. This figure was created using BioRender.com.

These are just some of the safety advantages of nucleic acid aptamers over proteins and small molecules, which are ideally beneficial in chronic musculoskeletal diseases. In most cases, chemically modified DNA and RNA aptamers do not integrate, replicate, or are excreted through the kidneys by renal clearance and degraded by heat and enzymes into low‐molecular‐weight metabolites, which are typically excreted into pools of [28]. Aptamers administered systemically or locally have been tested in preclinical studies and have shown a desirable overall safety profile with neovascular age‐dependent macular degeneration aptamer pegaptanib and some oncology or cardiovascular drugs in preclinical development (Figure 11) [28, 140]. The causes of adverse events are mostly related to the route of administration (intravitreal injection) or the conjugated chemistries, but not the backbone oligonucleotide [28, 140]. In this type of research, there are no homogeneous reports of genotoxicity, retarded organ toxicity, or off‐target tissue injury to the target of aptamer connectivity. This supports the idea that the key attributes of safety are the selection of targets, chemical modification, and carrier design, and not the intrinsic properties of nucleic acids.

### 8.2 Carrier and Payload Toxicities

The carrier and payload may be of greater concern than the aptamer in musculoskeletal applications. When the surface charge, size, or excipient composition of lipid‐based and polymeric nanoparticles is not optimized, they can cause dose‐limiting hepatic or hematologic toxicity, complement activation, and infusion reactions [94, 107, 141, 142, 143]. They can destabilize cell membranes and cause inflammatory processes and are unwarranted in inflamed joints with osteoarthritis or rheumatoid synovitis. Hydrogels and DNA nanostructures are generally biocompatible in vivo. However, their degradation kinetics, crosslinkers, and osteoinductive components incorporated into the biomaterial should be carefully selected to prevent the formation of chronic foreign‐body reactions or ectopic calcification of bone and cartilage tissues [107].

The second safety measure is the selection of the payload. There is a potential risk of local benefit and systemic exposure to cytotoxic chemotherapeutics, ICD inducers, and high‐potency biologics due to target failure or depot leakage. Aptamer‐based systems, in which bone tumors are targeted with doxorubicin or photosensitizers, reduce systemic exposure compared to free drugs; however, they should be dose‐escalated, cardiac‐monitored, and secondary malignancies and marrow‐monofiltrage should be observed in the long term [28, 99].

Likewise, the doses of antimicrobial payloads delivered to osteomyelitis structures must be sufficient to kill biofilms without resistance and without damaging osteoblast function. In practice, musculoskeletal aptamer‐nanocarrier preclinical safety testing should include both conventional nanotoxicology outcomes (hematology, serum chemistry, and organ histology) and bone and joint outcomes (cartilage integrity, bone turnover markers, and marrow cellularity).

### 8.3 Immunogenicity: Anti‐PEG, Anti‐nucleic Acid, and Complement Activation

Immunogenicity is a key issue for any aptamer‐functionalized nanocarrier intended for repeated use in chronic illnesses, such as osteoporosis, osteoarthritis, or RA. Although modified or suitably modified nucleic acids are typically less immunogenic than most biologics, the sequence motifs of unmethylated CpG or other double‐stranded regions may stimulate toll‐like receptors and innate immunity, particularly when administered systemically in large doses [28]. These immune responses can be reduced by the careful design of sequences and selective use of 2^′^‐O‐methyl, 2^′^‐fluoro, or locked nucleic acid (LNA) residues without affecting target affinity [107, 144].

However, immunity against stealth polymers, especially poly (ethylene glycol) (PEG), is well characterized. Anti‐PEG antibodies exist in a large number of patients and have been associated with accelerated blood clearance, infusion reactions, and infrequent severe hypersensitivity events when using most PEGylated drugs [95, 111, 145, 146].

PEGylated RNA aptamer‐based REG1 anticoagulation also failed in the REGULATE‐PCI trial, showing that PEG‐related anaphylactoid reactions were the main issue and that the oligonucleotide itself was not the cause of the reaction [147]. Systematic screening of anti‐PEG antibodies, testing of alternative stealth coatings (such as zwitterionic or polysarcosine‐based polymers), and a comprehensive examination of complement activation‐related pseudoallergy (CARPA) should be considered important in musculoskeletal candidates who might need a long‐lasting dose, potentially due to the tendency to form biofilms [107, 111, 148]. The complement system and innate immunity can also be activated on nanoparticle surfaces, regardless of the presence of PEG. High‐density aptamer coating, specific lipid and polymer compositions, and opsonization can be stimulated, leading to rapid clearance and infusion‐related reactions [107, 142, 143, 149].

Thus, early‐stage musculoskeletal interventions must include human serum complement testing, cytokine release testing, and, when available, ex vivo testing of synovial fluid or bone marrow aspirate from patients with inflammatory diseases. In theory, these interdependent safety areas, aptamer sequence, chemical modification, stealth polymer, carrier surface, and payload are more appropriately considered as a set, as presented schematically in the safety‐immunogenicity ‘wheel’ (Figure 11).

### 8.4 Local Tissue Compatibility in Bone, Cartilage, Marrow, and Synovium

Systemic tolerability does not guarantee safety in bone and joint tissues, which have unique mechanical, vascular, and immune environments. Intraarticular formulations must avoid chondrotoxicity, synovial irritation, and mechanical disruption of lubrication. Several viscosupplements and local anesthetic formulations approved for joint use have shown dose‐dependent chondrotoxic effects; aptamer‐decorated nanoparticles and hydrogels must meet at least the same standards [107, 150, 151].

For cartilage‐targeted systems, particles smaller than 100 nm are typically required to penetrate the dense extracellular matrix; however, overly cationic or rigid carriers may nonspecifically bind to proteoglycans, disrupt the osmotic balance, and damage matrix integrity [28, 152].

In the bone and marrow, local deposits placed in defects or along osteotomy lines must balance osteoinduction with the maintenance of normal remodeling and hematopoiesis. Highly crosslinked or slowly degrading scaffolds can lead to fibrous encapsulation or chronic inflammation, whereas excessively fast‐degrading systems may release their payloads too quickly, jeopardizing their mechanical stability [153]. For synovial and periarticular applications in RA and spondyloarthritis, existing chronic inflammation and immune dysregulation are of concern; even mild off‐target activation of innate immunity by carriers or chemistries can worsen the disease. Therefore, local safety assessment should go beyond basic histology to include standardized scoring of cartilage damage (such as OARSI systems), pannus and erosion assessments, and evaluation of bone microarchitecture, along with exploratory transcriptomic or cytokine profiling of the affected tissues [118, 153, 154].

### 8.5 Long‐Term and Repeat‐Dose Safety in Chronic Musculoskeletal Disease

Most clinical musculoskeletal features, including osteoporosis, osteoarthritis, RA, chronic osteomyelitis, and bone metastasis, warrant repeated or prolonged treatment. Thus, safety testing of aptamer‐functionalized nanocarriers should consider short‐term tolerability, cumulative exposure, immune memory, and possible tissue accumulation. PEG‐specific reactions and complement activation deteriorate with repeated doses, particularly when the dose intervals match those of anti‐PEG antibody kinetics [95, 111, 143, 147, 155, 156]. Similarly, completely nonbiodegradable nanoparticles are likely to accumulate in the liver, spleen, or bone marrow over time, and their impact on hematopoiesis and systemic immunity is unknown [19, 157, 158]. A stepwise approach should be taken with first‐in‐human musculoskeletal studies: local or single‐compartment delivery (such as intraarticular or depot injections in defect beds) at doses significantly lower than preclinical no‐observable adverse effect is advisable, with a later progressive follow‐up of musculoskeletal function, bone and cartilage imaging, and basic immune surveillance, and with predetermined criteria to stop should anaphylaxis develop [145, 159]. Repeat‐dose regimens may be raised incrementally with accumulating clinical experience, highlighting the combinations of chemically conservative aptamers, biodegradable carriers, and established payloads. At the same time, standardized safety reporting as mandated by the standards of nanomedicine and rheumatology/orthopedic trials will be needed to support cross‐program learning, as well as to optimize the safety and immunogenicity profile of next‐generation musculoskeletal aptamer therapies [87, 107, 159, 160, 161, 162].

## 9 Translation and CMC Manufacturing

**FIGURE 12:**
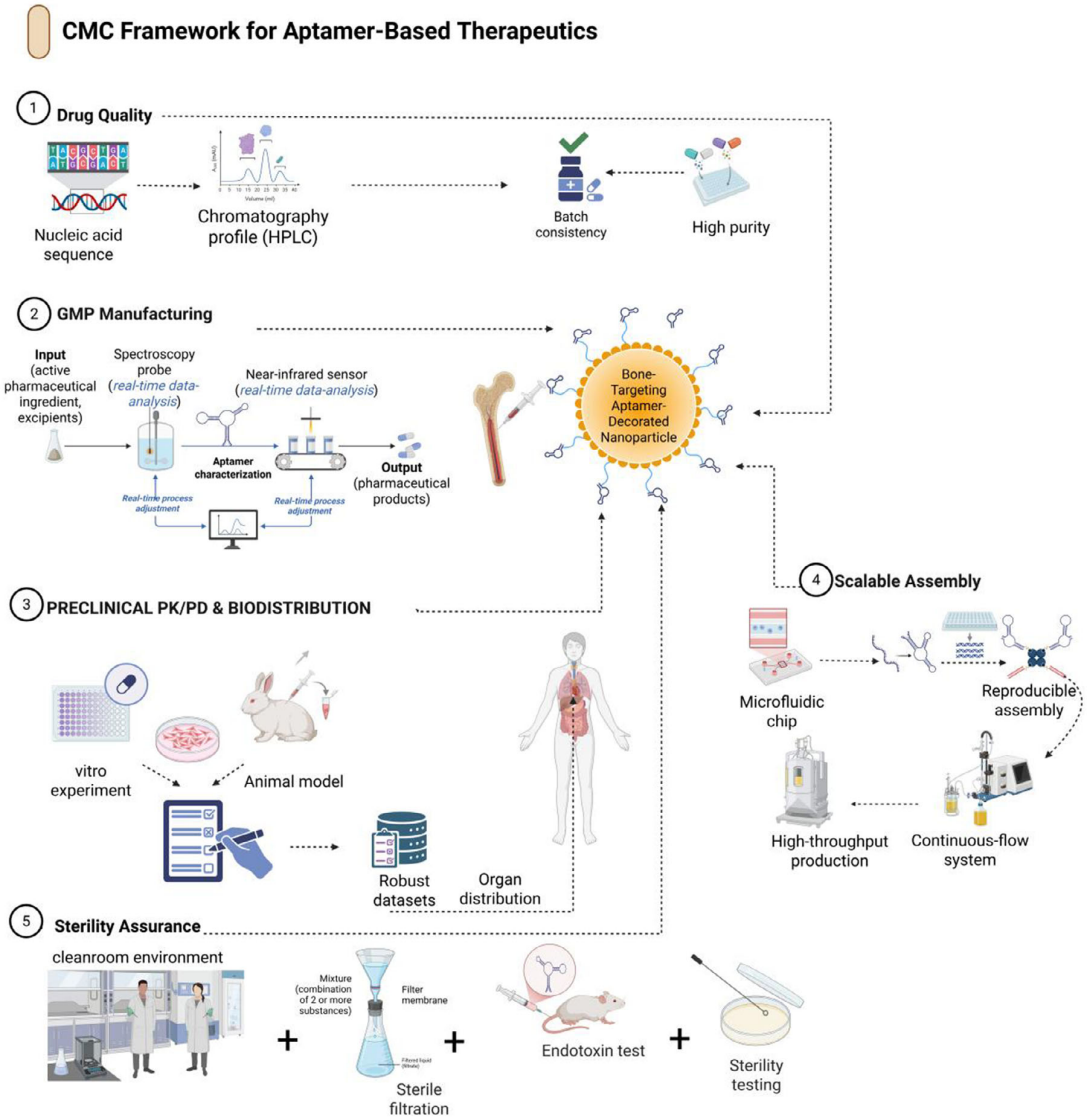
CMC and manufacturing practices for the clinical translation of aptamer‐based therapeutics. Radial schema summarizing chemistry, manufacturing, and control (CMC) requirements for clinically viable aptamer platforms. The central illustration depicts an aptamer‐decorated nanoparticle engineered for bone targeting. The surrounding sectors highlight drug quality (high identity and purity to minimize batch‐to‐batch variability and exclude animal‐derived contaminants), GMP synthesis and analytics (scalable, reproducible production, and quantitative assays confirming aptamer copy number per carrier and ligand integrity), scalable assembly methods (microfluidic or other advanced technologies enabling high‐throughput, clinically relevant manufacturing), preclinical pharmacokinetic/pharmacodynamic and biodistribution studies (robust datasets in small and large animals to support investigational new drug applications), and environmental controls and sterilization (validated aseptic processing, sterilization procedures, and environmental monitoring to prevent microbial contamination). Manufacturing considerations are informed by the analytical and regulatory requirements outlined for nucleic acid therapeutics, including aptamer identity and purity standards, scalable conjugation chemistry, and stability testing under conditions relevant to musculoskeletal product storage and distribution. This figure was created using BioRender.com.

Aptamer‐functionalized nanocarriers are to be applied synthetically in first‐in‐human applications and will rely on biological functionality as much as on chemical, manufacturing, and control (CMC) functions. Unlike proteins, oligonucleotide aptamers synthesized in solution are chemically synthesized and are thus highly standardized. However, the addition of carrier structures, stealth polymers, and bioactive payloads introduces complexities in advanced nanomedicines and combination products (Figure 12).

### 9.1 Analytical Regulations for Engineered Aptamers

At the aptamer level, production may be based on automated solid‐phase phosphoramidite chemistry, and the identity of the sequence and the correct location of functional groups (thiols, azides, and amines) or 2 to 2^′^‐F /2 to 2 the F /O methyl and heterobases can be checked in‐line or in batch [28, 163]. Identity (LC‐MS, HPLC), purity (usually 90%–95%), counter‐ion content, residual solvents, and confirmation of an interesting aspect of quality, such as binding affinity and secondary structure in physiological ionic conditions, are the main release tests [28, 163]. The user must establish that aptamer conformation and target binding are maintained in the appropriate matrices for bone and joint diseases, such as serum, synovial fluid, and marrow supernatant, rather than simply in buffers, to apply aptamers to musculoskeletal applications.

### 9.2 Scalable Aptamer Nanocarrier Platform Development

Following the interactions during GMP aptamer synthesis, scalable aptamer–nanocarrier assembly is the major challenge in CMC. The process parameters (such as the solvent formulation structure, microfluidic mixing flow rates, and downstream processing operations such as filtration and concentration of the solvent) will be optimized to yield a desired size (typically between 50–150 nm), low polydispersity index (PDI < 0.2), and a desired distribution of surface charges [107, 164, 165] in the case of lipid and polymeric nanoparticles. One such feature is the aptamer density, which is particularly sensitive to aptamer‐decorated systems; low density causes inefficient targeting, whereas high density may cause steric crowding or aggregation [76, 107]. To achieve this, batch‐release assays need to measure the number of aptamer copies per particle (such as by fluorescent labeling, isotope detection, or surface plasmon resonance) and compare the results with in vitro binding and cell uptake.

This is also true for dendrimers, DNA nanostructures, and hybrid carriers. The direction of DNA nanostructures and hydrogel manufacturing is connected with the probing folding, the level of crosslinking, and the size of the mesh, which influence the mechanical properties and release dynamics [28, 107]. Additional disparities in biological applications constitute exosome and extracellular vesicle platforms: the upstream cell culture, vesicle isolation, and aptamer decoration must be in time with each other to ensure a stable vesicle size, cargo, and exposure of surface ligands [107, 109, 166].

### 9.3 Distribution of Complex Products

Most engaging musculoskeletal designs, such as aptamer–GelMA hydrogels to repair osteoporotic defects, aptamer‐functionalized meniscal scaffolds, aptamer‐decorated microneedle arrays, and exosome‐based depots in the articular space, are inexplicably multicomponent. In practice, comanufacturing is likely to be more of a modular form: (i) GMP manufacturing of the aptamer; (ii) GMP or high‐grade manufacturing of a carrier (nanoparticles, hydrogels, scaffolds, microneedles, exosomes); and (iii) a masterful conjugation or loading step that unites the two in conditions that have not been shown to alter aptamer and carrier integrity [107, 109].

### 9.4 Conditions of Stability, Sterilization, and Real‐Life use

Aptamer‐nanocarrier system stability programs that will be applied in bone and joint conditions must consider both standard pharmaceutical stressors and musculoskeletal‐specific conditions. Accelerated stability tests should be performed, and long‐term stability tests should be performed on physicochemical properties (size, PDI, zeta potential, and ligand density), aptamer stability (nuclease degradation, and depurination), and functional binding over time and at varying storage temperatures [28, 107]. To administer the products intravenously into the synovial fluid and serum, additional screening of human/animal synovial fluid and serum may indicate unexpected aggregation, opsonization, or premature aptamer disaggregation [87]. Sterilization is one of the issues. These polymeric nanoparticles, DNA nanostructures, and hydrogels cannot withstand the high temperatures of autoclaving or high gamma radiation. Any such event that can be filtered would preferably undergo aseptic processing and sterile filtration to regulate the aptamer structure, ligand density, and mechanical properties. Scaffolds, microneedles, and implants would avoid filtration to low‐dose gamma or electron‐beam irradiation or ethylene oxide treatment [49, 104, 167]. For musculoskeletal implants at load‐bearing sites, it may be necessary to test the device under additional mechanical fatigue testing under physiological conditions to ensure that sterilization does not affect the device's long‐term performance.

### 9.5 Regulatory Aspects of the use of Multicomponent Aptamers as Nanomedicals

Regulators have been requesting that sponsors of nanomedicines report on their products in full, specifying the material properties that are significant for biological functionality and clinical safety. For aptamer‐nanocarriers, this means adopting a quality‐by‐design approach, which involves identifying the quality attributes of critical (CQA), including aptamer sequence and modification pattern, particle size and PDI, ligand density, and payload loading, and correlating them with critical process parameters [30, 159, 168, 169, 170]. The reflection paper on nanotechnology‐based medicinal products published by the European Medicines Agency and the guidance provided by the U.S. The Food and Drug Administration on drug products containing nanomaterials both reveal that a sufficient characterization of structure, aggregation, surface properties, and in vitro release behavior is essential as a sufficient first step prior to meaningful in vivo studies.

This is because, in combination products such as aptamer‐functionalized scaffolds or microneedle devices, developers must fulfill the requirements of biocompatibility, sterilization, and mechanical performance, in addition to the requirements of medicinal‐product standards in pharmacology, toxicology, and pharmacokinetics. Musculoskeletal aptamer programs. Regulators should note that musculoskeletal aptamer programs will have the quickest prospect of evolving when they acknowledge regulatory historic annexes to present nanoliposomal medications, depot corticosteroids, and step‐premo boiler house‐ones, and restructure their CMC packages to manage the expectations of both small‐molecule/nucleic‐acid regulating bodies and device regulators [107].

## 10 Clinical and Regulatory Prognosis

### 10.1 Value of First‐in‐Human Indications

The present clinical experience with systemic aptamer‐based therapeutics, which comprise the majority of existing clinical aptamers (VEGF‐binding aptamer pegaptanib for neovascular age‐related macular degeneration and CXCL12‐binding aptamer olaptesed pegol for hematologic malignancies), has demonstrated that chemically modified aptamers can achieve acceptable safety profiles and predictable pharmacokinetics in humans [23, 140, 171]. Signs of the musculoskeletal system will present earlier in the clinic for local or regionally specific applications (where the risks are much easier to manage) and in cases where the pharmacodynamic expression is limited to the anatomical field.

It is possible to draw out three early targets. To treat intraarticular osteoarthritis, aptamer‐functionalized hydrogels or nanoparticles with either anti‐catabolic or pro‐anabolic molecules can be delivered intraarticularly, and the delivery location is accessible and provides access to the disease site and specific imaging and clinical outcomes (pain, function, and MRI cartilage morphology) [172]. Second, the bone defect systems contain local bone defect systems targeting high‐risk osteoporosis or traumatic defects (such as CH6‐like osteoblast‐specific depots in mandibular or long‐bone defects), to which already known systems of bone graft substitutes and growth factor‐impregnated scaffolds already possess regulatory precedents [47, 173]. Thorthopedicology and orthopedic crosses, systemic bone metastasis: here, bone‐homing aptamer‐nanocarriers can first be evaluated as an imaging modality or as a delivery cargo in high‐risk settings by taking advantage of systemic tolerance in oncology [87, 100, 130].

### 10.2 Prototype Phase I/II Design Trials

A one‐knee, intraarticular aptamer‐hydrogel depot of chondrocytes or synovial fibroblasts may be one such Phase I/II trial in OA, whereby the primary endpoints may be safety (local reactions, infection, effusion) and pharmacokinetics in synovial fluid, and secondary endpoints can include pain, function, and quantity of MRI or radiographic data of cartilage structure (6–12 months) [172, 174]. In cases such as osteoporosis or segmental defects, where aptamer‐modified bone‐regeneration scaffolds could be installed into either the mandibular or metaphyseal defect of already surgically compromised patients, safety and local integration would be the primary outcomes, with uCT, BMD, and biomechanical surrogates as the secondary outcomes [175, 176].

Bone metastases, both diagnostic and theranostic constructs, such as bone‐homing aptamer‐nanoparticles of imaging agents or low‐dose cytotoxic agents, can initially be diagnostic or theranostic, for example, to measure biodistribution, bone: RES ratios, and preliminary antitumor activity in patients with advanced prostate or breast cancer in the bone skeletal disease model [87, 177]. In all cases, dose‐escalation plans ought to be reasonable, and special care must be taken to note the drug's immunogenicity, off‐target organ toxicity, intraarticular events, and progressive joint atrophy.

### 10.3 Problems with Regulatory Pathways and Combination Products

Most aptamer nanocarrier systems used to treat musculoskeletal disorders are classified as combination products comprising a drug, a device, and, in some cases, a portion of a biologic. Intraarticular aptamer‐hydrogel to be a drug‐device combination product; an aptamer‐functionalized scaffold or microneedle array can be a target of device regulation with a drug constituent; exosome‐based systems will also prompt other biologic classification issues [178, 179]. The right interactions with regulators to clarify the main mode of action, product classification, and preclinical packages had to be established earlier to avoid delays at an advanced stage.

Regulatory provisions for nanomaterials are based on the information that CMC data should be related to in vivo performance. Both the European Medicines Agency and U.S. The Food and Drug Administration highlights that nanomaterial structure, surface characteristics, aggregation, and release kinetics must be described in detail, with proper in vitro and in vivo models that reflect the proposed clinical use being provided [178]. In musculoskeletal aptamer biosensors, this means that not only generic tumor xenografts or back depots but also bone‐ and joint‐relevant models must be employed at an early stage of development. Biomarkers and Radiographic Outcomes. The translational method is to use biomarkers and imaging of the readout to monitor the deficiencies and disruptions of cellular and tissue physiology, as well as the severity of the disease [174, 180].

Finally, successful clinical translation is anchored in robust biomarkers and imaging modalities that can assess targeting performance and treatment response. Quantitative CT, high‐resolution peripheral CT, and MRI are used to examine bone mass and microarchitecture in detail [175, 176]. Compositional MRI (T2, T1r, and dGEMRIC) and normative radiographic scoring are emerging as outcomes for cartilage and meniscus [174, 181]. Synovial reactivity will be evaluated using imaging techniques (ultrasound and MRI) and cytokines in the clinical scoring of inflammatory arthritis. In bone tumors and infections, volumetric imaging and bone integrity measurements are necessary (with functional imaging, where necessary (that is, PET tracer to assess infection or tumor metabolism) [87, 182, 183].

**FIGURE 13:**
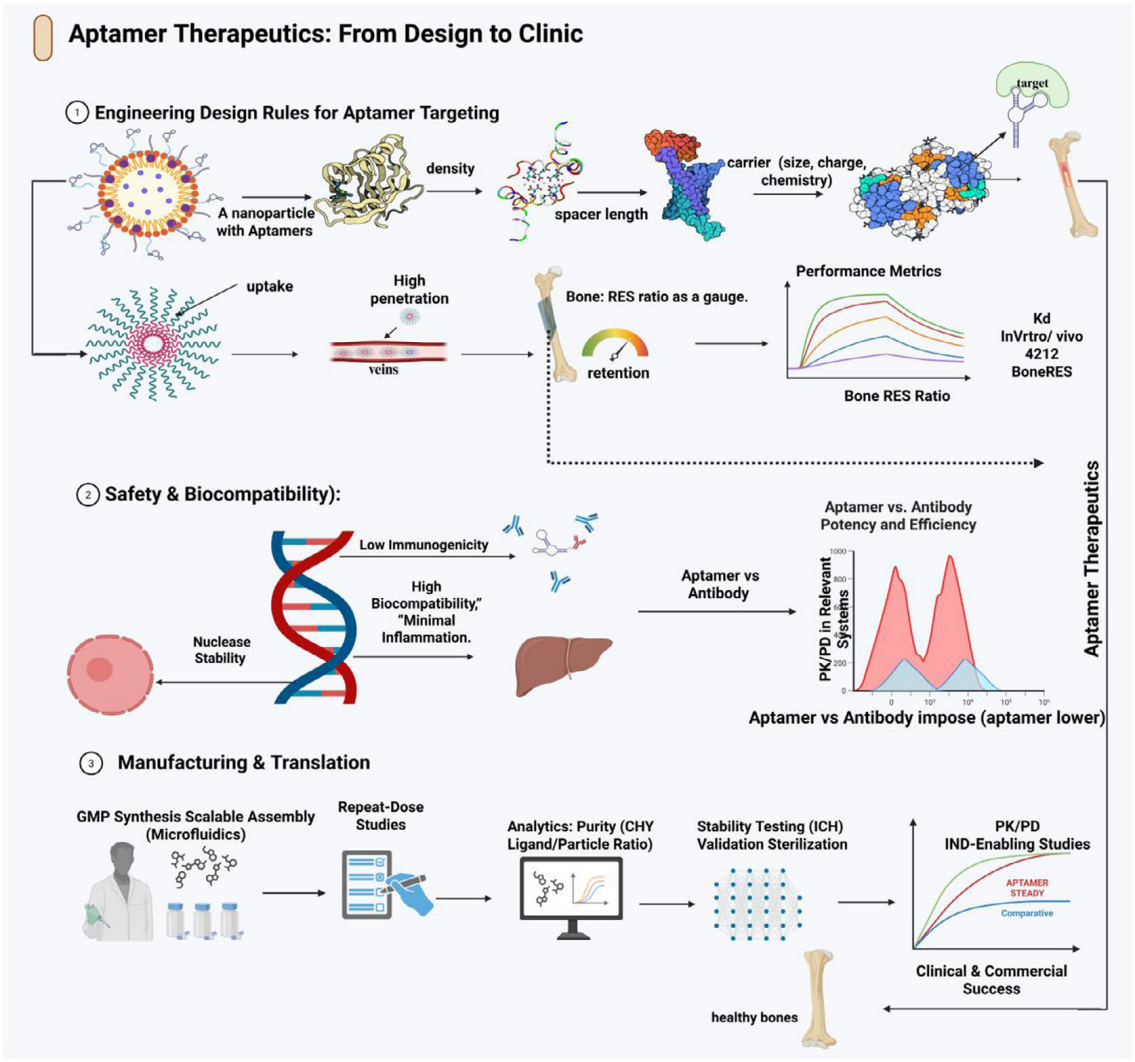
Aptamer therapeutics: from design rules to clinical deployment. Translational roadmap for aptamer‐based therapeutics in musculoskeletal diseases. The upper tier focuses on engineering design rules, including the control of carrier type, size, charge, aptamer density, spacer length, and binding kinetics, with bone‐to‐reticuloendothelial system ratios used as performance gauges. The middle tier addresses safety and biocompatibility, emphasizing nuclease stability, low immunogenicity, high biocompatibility, and favorable inflammatory profiles, and benchmarking aptamers against antibodies in terms of potency and pharmacokinetics/pharmacodynamics. The lower tier outlines the manufacturing and translation steps, from scalable GMP synthesis and repeat‐dose toxicology through analytical characterization (ligand‐per‐particle ratio, purity), stability testing, and sterilization to IND‐enabling PK/PD studies. The successful execution of this continuum underpins the clinical and commercial deployment of aptamer therapeutics and improved bone and joint outcomes. Priority first‐in‐human indications include intraarticular delivery for osteoarthritis, defect‐bed implantation for bone regeneration [184], and systemic targeted delivery for skeletal metastases [53], each with measurable clinical endpoints and existing standards of care for comparison. This figure was created using BioRender.com.

Engineeringly, such readouts are not results; they will be control signals that guide iterative processes such as aptamer selection, ligand density, carrier geometry, and dose scheduling and will establish a feedback loop between the design idea, CMC, and clinical outcome. The musculoskeletal aptamer arena is thus poised to capitalize on the intimate fit between manufacturing, regulation, and biomarker‐driven trial design to transform reliance on an already vast array of preclinical units into viable first‐in‐human candidates (Figure 13).

## 11 Open Problems and Future Directions

Nanocarriers with aptamers and functional groups targeting musculoskeletal diseases have been developed in isolation. Tables 1 and 2 display the case studies and aptamer panels, respectively, which demonstrate what can be achieved. Nevertheless, they also expose the field's structural problems: a lack of coherent characterization, multiple models, and minimal consensus on shared benchmarks. The future must translate stimulating prototypes into viable clinical platforms through standardization, deeper target discovery, and integrated translational infrastructure, rather than single, rudimentary concepts.

### 11.1 Characterization and Reporting Standardization

A persistent limitation in the literature is the incomplete reporting of critical design variables. Affinity is commonly reported as high, without an effective quantification of the dissociation constant under physiological conditions. Aptamer‐modified nanoparticles have also been reported without descriptions of ligand density, spacer chemistry, orientation, particle size, PDI, or zeta potential; however, biodistribution outcomes, such as bone‐to‐RES ratios or joint retention, are rarely reported. The measurement of the outcomes of bone, cartilage, synovial tissue, tumors, and infections on the biological side is performed using devices similar to those above; however, protocols and scoring systems are often changed, shortened, or not described in detail.

The most viable solution is to adopt a minimal reporting set. At the physicochemical level, any aptamer–nanocarrier system intended for translational application must provide Kd (under assay conditions), aptamer copies/particle or surface area, spacer/linker nature and length, and core particle parameters, such as size, PDI, and zeta potential. At the biological level, community standards of imaging, histology, biomechanics, tumor, and infection should be used to record outcomes in bone, cartilage/meniscus, synovium, tumor, and infection, where available. In their absence, there will be no meaningful cross‐platform comparison and no real structure–function analysis (Figure 10).

### 11.2 Discovery of the Target and Model Relevance

The present musculoskeletal aptamer library includes obvious targets, such as osteoblasts, stromal cells, chondrocytes, synovial cytokines, cancer stem cell markers, and bacterial epitopes. Although this represents a logical starting point, it may overlook the diversity of bone and joint microenvironments. Currently, single‐cell and spatial omics are mapping the cellular and molecular heterogeneity of bone, cartilage, synovium, and marrow in unprecedented detail; these datasets should be subjected to extensive analysis to identify disease‐specific epitopes for SELEX and in vivo SELEX, rather than relying on classical markers. The model choice must evolve along with the other processes. There are numerous systems today based on small animals with defects or disease models that do not fully recapitulate human anatomy, loading patterns, and immunological responses. The implementation of in vivo SELEX, supported by validation experiments in orthotopic, load‐bearing, and infection‐prone sites, will be important to ensure that the selected aptamers remain functional under the complex conditions of human‐like skeletal physiology.

### 11.3 Designs Based on Logic Gating and Multiaptamers

To date, the vast majority of musculoskeletal platforms have used a single aptamer on the same carrier. This limits the information‐processing capacity of nucleic acid architecture. Logic‐gated and multiaptamer designs have been established in oncology and biosensing, and the next logical step in bone and joint applications is to use logic gates. Therefore, dual‐aptamer systems must recognize a structural signal (such as a mineralized matrix) and a disease‐related receptor simultaneously to improve discrimination between diseased and healthy tissues. Moreover, OR/NOT logic can be used to block uptake in tissues that express a set of markers for a specific do‐not‐target or in microenvironments with a specific signal combination. Such architectures have significantly higher demands for controlling the ligand density, spatial structure, and conformational dynamics than typical. Their incorporation into musculoskeletal models will require not only creative design but also cautious estimation of the effects of logic‐gated constructs on biodistribution, off‐target uptake, and therapeutic indices relative to single‐ligand analogs.

### 11.4 Current Limitations of Aptamer‐Nanocarrier Systems in Musculoskeletal Applications

Despite encouraging preclinical results, several critical limitations must be acknowledged. First, aptamer stability remains a concern in musculoskeletal microenvironments. Although chemical modifications improve nuclease resistance, the long‐term functional integrity of aptamer–nanocarrier constructs in protease‐ and nuclease‐enriched synovial fluid, acidic bone resorption lacunae, and biofilm‐associated infection sites has not been systematically characterized under clinically relevant exposure durations.

Second, the formation of a protein corona upon systemic administration can mask surface‐displayed aptamers, reduce effective ligand availability, and alter biodistribution in ways that are difficult to predict from in vitro binding assays alone. This phenomenon is rarely quantified in musculoskeletal aptamer studies; nevertheless, it may substantially affect targeting efficiency in vivo.

Third, almost all current evidence is derived from small animal models that do not fully recapitulate the mechanical loading, anatomical scale, vascular architecture, or immunological complexity of human bone and joint tissues. The absence of validated large‐animal or humanized models limits the predictive value of the reported efficacy and biodistribution data for clinical translation.

Fourth, no aptamer‐functionalized nanocarrier has entered clinical trials for any musculoskeletal indication. The field lacks head‐to‐head comparisons with clinically established targeting ligands, such as antibodies or bisphosphonates, under equivalent conditions, making it difficult to assess the true competitive advantage of aptamer‐based systems.

Fifth, manufacturing scalability presents unresolved challenges. The batch‐to‐batch reproducibility of aptamer conjugation efficiency, ligand orientation, and nanocarrier physicochemical properties has not been demonstrated at scales required for clinical development. The regulatory pathway for multicomponent aptamer–nanocarrier products remains undefined, adding uncertainty to translational timelines.

These limitations do not diminish the potential of aptamer‐guided delivery but underscore the need for rigorous standardized evaluation before clinical advancement can be justified.

### 11.5 AI‐Assisted SELEX and Computational Aptamer Design

Machine learning and deep learning are beginning to transform aptamer discovery. Computational tools, such as AptaDiff, RaptGen, and Apta‐MCTS, can generate de novo aptamer sequences with predicted high‐affinity binding, thereby bypassing the iterative cycles of traditional SELEX. Notably, an interactive machine learning approach applied to the post‐SELEX chemical modification of an anti‐sclerostin aptamer achieved a 10^5^‐fold enhancement in binding affinity, directly demonstrating the relevance of AI‐guided optimization for musculoskeletal targets. The integration of these computational strategies with microfluidic and HTS SELEX platforms could dramatically accelerate the identification of aptamers against underexplored skeletal epitopes identified through single‐cell and spatial omics.

### 11.6 Multifunctional DNA Nanostructures for Targeted Bone and Joint Therapy

DNA origami and other programable DNA nanostructures offer precise spatial control over ligand presentation, drug‐loading stoichiometry, and stimulus‐responsive release. Their programmability enables the construction of multivalent, multiaptamer architectures that simultaneously engage cellular targets and matrix components, a capability particularly suited to the heterogeneous microenvironments of bone defects, articular cartilage, and inflamed synovium. Although DNA nanostructures have been explored in oncology and biosensing, their systematic application to musculoskeletal indications remains nascent and represents a promising frontier for next‐generation aptamer‐guided drug delivery.

### 11.7 Aptamer‐Guided Theranostics and Combination with Immunotherapy or Cell‐Based Therapies

Theranostic strategies that combine aptamer‐mediated targeting with imaging functionality, such as fluorescence, photoacoustic, or magnetic resonance contrast, could enable real‐time monitoring of drug biodistribution and therapeutic responses in bone and joint tissues. Furthermore, integrating aptamer‐functionalized nanocarriers with emerging immunotherapeutic or cell‐based approaches, including macrophage polarization strategies, chimeric antigen receptor T‐cell therapies for osteosarcoma, and mesenchymal stromal cell codelivery, may yield synergistic effects that single‐modality aptamer systems cannot achieve independently.

### 11.8 Clinical Translation and Industrialization Pathways

Accelerating the clinical translation of aptamer‐nanocarrier systems will require coordinated efforts across several fronts: establishment of GMP‐compliant manufacturing processes with validated batch‐to‐batch reproducibility, development of companion diagnostic assays to identify patient populations most likely to benefit from aptamer‐guided therapy, and engagement with regulatory agencies to define classification and approval pathways for multicomponent aptamer products. High‐priority first‐in‐human indications, including intraarticular delivery for osteoarthritis, defect‐bed implantation for bone regeneration, and systemic targeted delivery for skeletal metastases, should be advanced through adaptive clinical trial designs that incorporate pharmacokinetic endpoints, imaging‐based biodistribution readouts, and standardized clinical outcome measures.

## 12 Conclusions

Aptamer‐functionalized nanocarriers offer a rational and adaptable platform for targeted intervention in musculoskeletal diseases, with growing evidence supporting their use in osteoporotic defects, cartilage and meniscal repair, inflamed synovium, bone tumors, and prosthetic joint infections. These ligands provide three essential advantages: programable affinity and selectivity, tunable chemical stability, and modular integration with various delivery vehicles. Despite this promise, most current systems remain prototypes, with inconsistent reporting of critical design parameters, such as binding affinity, ligand density, spacer chemistry, and carrier physicochemical properties. To transition from proof‐of‐concept to clinically viable therapies, these variables must be rigorously defined, coupled with standardized biodistribution, histologic analyses, and comprehensive safety evaluation in mechanically and tissue‐relevant models. Once these benchmarks are met, translation becomes a matter of performance. Local depots for osteoarthritis and meniscal repair, defect‐bed systems for osteoporotic or traumatic bone loss, and bone‐targeted carriers for skeletal metastases represent the most realistic first‐in‐human applications, each with measurable outcomes and existing standards of care for comparison. The future of the field hinges not on new conceptual breakthroughs but on operational execution, including engineering aptamers that withstand the distinct challenges of bone, cartilage, synovium, and marrow, and delivering them through reproducible, quantitatively defined platforms that impact therapeutic decision‐making and musculoskeletal health outcomes.

## Funding

This study was supported by National Natural Science Foundation of China (32571692).

## Conflicts of Interest

The authors declare no conflicts of interest.

## Data Availability

Data sharing not applicable to this article as no datasets were generated or analysed during the current study.
